# Large-scale death of retinal astrocytes during normal development is non-apoptotic and implemented by microglia

**DOI:** 10.1371/journal.pbio.3000492

**Published:** 2019-10-18

**Authors:** Vanessa M. Puñal, Caitlin E. Paisley, Federica S. Brecha, Monica A. Lee, Robin M. Perelli, Jingjing Wang, Emily G. O’Koren, Caroline R. Ackley, Daniel R. Saban, Benjamin E. Reese, Jeremy N. Kay

**Affiliations:** 1 Department of Neurobiology, Duke University School of Medicine, Durham, North Carolina, United States of America; 2 Department of Ophthalmology, Duke University School of Medicine, Durham, North Carolina, United States of America; 3 Department of Immunology, Duke University School of Medicine, Durham, North Carolina, United States of America; 4 Neuroscience Research Institute, University of California, Santa Barbara, Santa Barbara, California, United States of America; 5 Department of Cellular, Molecular and Developmental Biology, University of California, Santa Barbara, Santa Barbara, California, United States of America; 6 Department of Psychological and Brain Sciences, University of California, Santa Barbara, Santa Barbara, California, United States of America; University of Basel, SWITZERLAND

## Abstract

Naturally occurring cell death is a fundamental developmental mechanism for regulating cell numbers and sculpting developing organs. This is particularly true in the nervous system, where large numbers of neurons and oligodendrocytes are eliminated via apoptosis during normal development. Given the profound impact of death upon these two major cell populations, it is surprising that developmental death of another major cell type—the astrocyte—has rarely been studied. It is presently unclear whether astrocytes are subject to significant developmental death, and if so, how it occurs. Here, we address these questions using mouse retinal astrocytes as our model system. We show that the total number of retinal astrocytes declines by over 3-fold during a death period spanning postnatal days 5–14. Surprisingly, these astrocytes do not die by apoptosis, the canonical mechanism underlying the vast majority of developmental cell death. Instead, we find that microglia engulf astrocytes during the death period to promote their developmental removal. Genetic ablation of microglia inhibits astrocyte death, leading to a larger astrocyte population size at the end of the death period. However, astrocyte death is not completely blocked in the absence of microglia, apparently due to the ability of astrocytes to engulf each other. Nevertheless, mice lacking microglia showed significant anatomical changes to the retinal astrocyte network, with functional consequences for the astrocyte-associated vasculature leading to retinal hemorrhage. These results establish a novel modality for naturally occurring cell death and demonstrate its importance for the formation and integrity of the retinal gliovascular network.

## Abstract 2

A study of the neonatal mouse retina shows that developmental cell death of retinal astrocytes does not occur by apoptosis but is instead mediated by microglia, which kill and engulf astrocytes to effect their developmental removal.

## Introduction

Naturally occurring developmental cell death contributes to the histogenesis of most tissues [1,2]. For example, in the mammalian central nervous system (CNS), many populations of neurons are subject to large-scale death that eliminates as many as half of the cells originally produced during neurogenesis [3,4]. Cell removal on this scale has a profound impact on neuroanatomy and circuit structure, and is essential for key steps in CNS morphogenesis [5–8]. Thus, to understand the fundamental developmental mechanisms that sculpt circuit anatomy and function, it is critical to document the extent of naturally occurring CNS death and the mechanisms by which it occurs.

In mammals, naturally occurring cell death typically occurs via apoptosis during specific developmental periods [4]. Following death, professional phagocytes such as CNS microglia are recruited to clear apoptotic corpses. This sequence of events is critical for normal development, because mice lacking essential apoptotic genes exhibit a range of defects affecting the architecture of the CNS and the patterning of its cellular populations [4,5,9,10]. Autophagy and necroptosis are non-apoptotic mechanisms that can occur in a variety of contexts, but their contribution to mammalian CNS development is minimal [11,12]. However, death by other mechanisms could still make important contributions to CNS development [13].

It has recently emerged that CNS cell death can be mediated through engulfment and killing of viable cells [14]. In contrast to apoptosis, in which phagocytosis serves a debris-removal role, this variety of death involves phagocytosis prior to or concomitant with cell killing. As such, it may be termed “death by phagocyte.” Microglia have been shown to engage in phagocytic killing in pathological contexts [15–17] and in the developing mammalian forebrain, where they appear to eliminate live neural progenitors within the subventricular zone [18]. These observations raise the possibility that death by phagocyte might sculpt other developing CNS cell populations. However, this possibility remains to be tested.

While developmental death of neurons has been extensively studied for almost a century, few studies have examined the role of death in the development of astrocytes. Because astrocytes have many essential roles in neuronal and vascular physiology [19–22], it is critical to understand the histogenetic mechanisms—such as regulation of cell number—that shape astrocyte development. Apoptotic astrocytes have been reported in developing cortex, retina, and cerebellum [23–29], but there is little evidence that such death is extensive enough to change astrocyte number. It remains to be determined whether death sculpts the developing astrocyte population, and if so, what the death mechanism might be.

**Fig 1:**
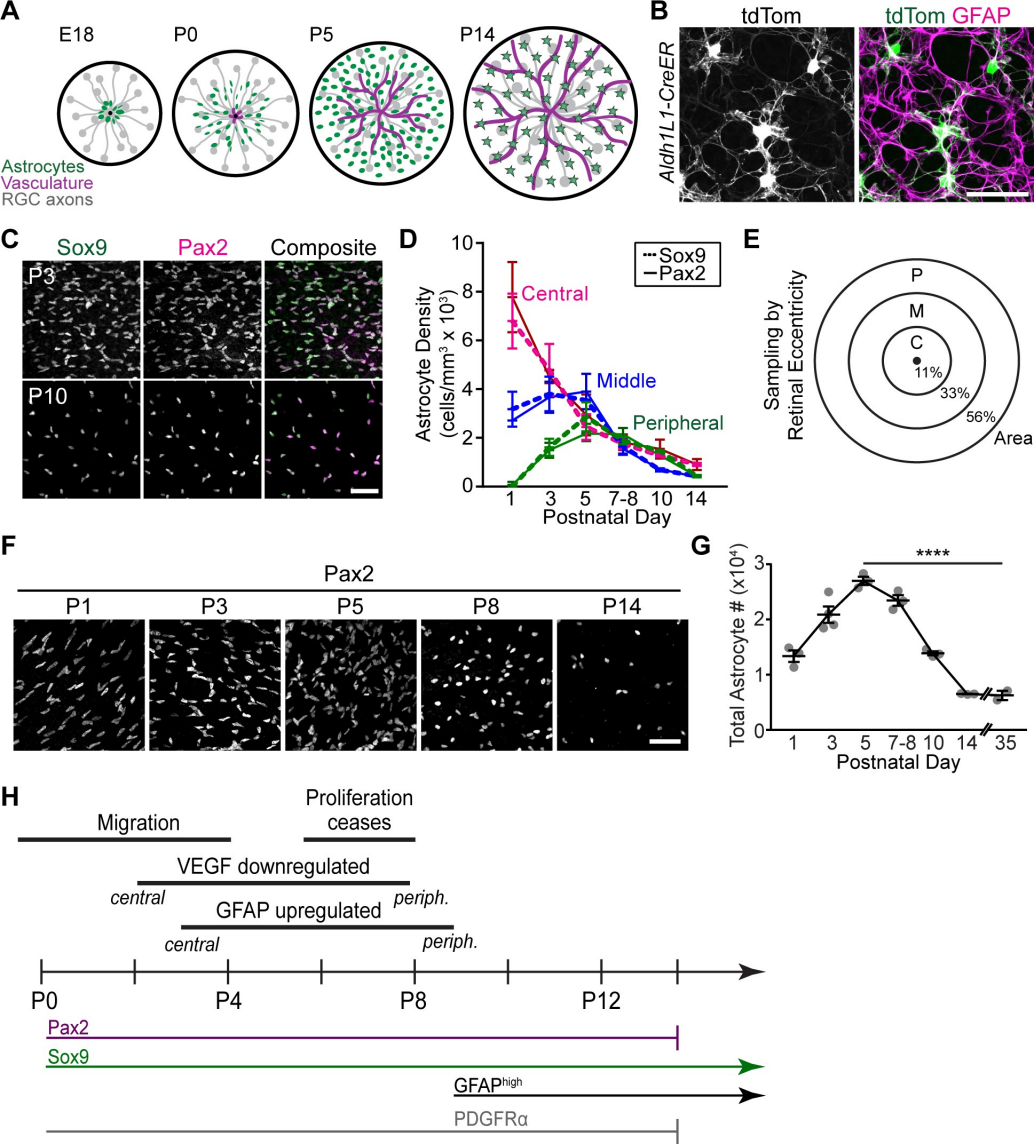
Developmental changes in astrocyte population size due to cell death. (A) Schematic of mouse RNFL development showing timing of retinal colonization by astrocytes and vasculature. Stars denote mature astrocytes. (B) Morphology of retinal astrocytes, revealed both by sparse genetic labeling (tdTom) and immunostaining of the full astrocyte network (GFAP). Astrocytes are shown en face, in confocal images of retinal whole-mounts. Aldh1l1-CreER mouse was used to drive sparse tdTomato expression. Similar to axon-associated astrocytes in brain, retinal astrocytes have a simple, fibrous morphology. (C-D) Two commonly used markers of retinal astrocytes, Sox9 and Pax2, label the complete astrocyte population across retinal development. (C) En face whole-mount views of RNFL. Pax2 and Sox9 colocalize in virtually all astrocytes. (D) Quantification of Sox9^+^ and Pax2^+^ astrocyte densities in central, middle, and peripheral retina. Density dynamics for each marker are nearly identical, suggesting that both markers label an identical population of cells across the developmental period studied here. Sample sizes: Pax2, *N* = 3 (P1, P5, P7–P8, P10, P14) or *N* = 4 (P3); Sox9, *N* = 2 (P1, P5), *N* = 3 (P10, P14), *N* = 4 (P7–P8), or *N* = 5 (P3). (E) Method to quantify total astrocyte number. Weighting was according to fraction of retinal area within each sampled region. (F) Astrocyte density, shown in en face images from retinal whole-mounts stained for Pax2. (G) Quantification of total astrocyte numbers across development, using retinas stained for Pax2 or Sox9. Statistics: one-way ANOVA followed by Sidak multiple comparisons test (see S1 Table for ANOVA details); ****p < 0.0001. (H) Time line of key developmental milestones (top) and marker expression (bottom) for retinal astrocytes. Immature astrocyte precursors are proliferative, express VEGF-A, and do not express high GFAP levels. Differentiation spreads in a center-to-peripheral wave roughly correlated with the spread of vascular wave front (A) [28,33]. High GFAP immunoreactivity is commonly used to demarcate astrocyte maturation. Note that death (C) continues to occur after astrocytes express mature phenotype by P10. Error bars: mean ± SD (D) or mean ± SEM (G). Sample sizes are depicted by data points on graph, except C,D. For data plotted in graphs, see S1 Data. Scale bar, 50 μm. C, central; GFAP, glial fibrillary acidic protein; M, middle; P, peripheral; PDGFR, platelet-derived growth factor receptor; RGC, retinal ganglion cell; RNFL, retinal nerve fiber layer; tdTom, tandem dimer Tomato; VEGF-A, vascular endothelial growth factor-A.

To investigate the scale of death during astrocyte development, we chose as our primary model system the astrocytes of mouse retina (Fig 1A and 1B). This axon-associated astrocyte population resides in a narrow stratum at the retinal surface, the retinal nerve fiber layer (RNFL), which consists primarily of astrocytes, blood vessels, and the axons of retinal ganglion cells (RGCs; Fig 1A). We chose these cells for two reasons. First, unlike brain astrocytes, they are confined to a monolayer within the RNFL, simplifying estimates of absolute cell number—a crucial advantage for studies of cell death. Second, regulation of astrocyte number may have implications for retinal function and disease. During angiogenesis, endothelial cells colonize the retina by using astrocyte arbors as an angiogenic patterning template [22]. When astrocyte numbers are experimentally elevated or lowered, both astrocyte and vascular patterning become disturbed, impairing vascular integrity [30–32]. It is therefore likely that astrocyte numbers are tightly developmentally regulated to prevent vascular pathology.

We find that retinal astrocytes are initially overproduced and then culled during a brief period of postnatal development. Surprisingly, apoptosis is not a major driver of their death; instead, we demonstrate that death by phagocyte plays a key role in this process. During the astrocyte death period, microglia interact extensively with astrocytes and engulf astrocytic material. These microglial behaviors are related to astrocyte killing rather than mere clearance of already-dead corpses, because ablation of microglia in vivo increased the number of viable, fully differentiated astrocytes. Finally, we find that astrocytic death by phagocyte can occur through either a heterotypic or a homotypic mechanism: when microglia are absent, astrocytes can partially compensate for their loss by eliminating each other. Together these data reveal that retinal astrocyte death has important mechanistic differences from other developmental deaths in the CNS. Thus, death by phagocyte might have broader roles in tissue morphogenesis than previously appreciated.

## Results

### Developmental changes in astrocyte population size due to cell death

To investigate whether retinal astrocytes have a period of naturally occurring cell death, we estimated total astrocyte number across postnatal mouse development. Estimates were generated from retinas stained in whole-mount for two well-characterized nuclear markers of retinal astrocytes, Pax2 or Sox9 (Fig 1C, 1D and 1H; [28]), using a sampling strategy described previously ([30]; Fig 1E). This analysis showed that retinal astrocyte numbers increased until P5, then declined substantially—by over 3-fold—to reach adult levels by P14 (Fig 1F and 1G). The rising phase of this curve is predominantly due to astrocyte migration into the retina, which is complete by P4–P5 ([22]; Fig 1A). Astrocyte loss was not due to migration out of the tissue, nor was it due to transdifferentiation into a non-astrocyte cell type, as shown by Cre-lox lineage tracing (S1A and S1B Fig). Moreover, the cell loss cannot be ascribed simply to down-regulation of the early astrocyte marker Pax2 (Fig 1H), as Sox9 gave identical results (Fig 1C and 1D). These results rule out the most likely alternative explanations for astrocyte disappearance, leading us to conclude that cell death is responsible.

To learn whether other astrocyte populations might also be subject to developmental death, we examined three other astrocytic cell types. Müller glia, the other astrocyte-like cell type of the retina, also showed a decline in cell number between early postnatal ages and adulthood. Similarly, in the brain, we observed developmental declines in density of protoplasmic astrocytes in the cerebral cortex and white-matter astrocytes in the corpus callosum (S2 Fig). Although not as definitive as the absolute cell number quantification that was possible in the retina, these observations suggest that brain astrocytes may also be overproduced and then culled. As RNFL astrocytes showed the largest magnitude of cell loss, we focused on this astrocyte population for subsequent investigation of death mechanisms.

### Retinal astrocyte death is not due to apoptosis

**Fig 2:**
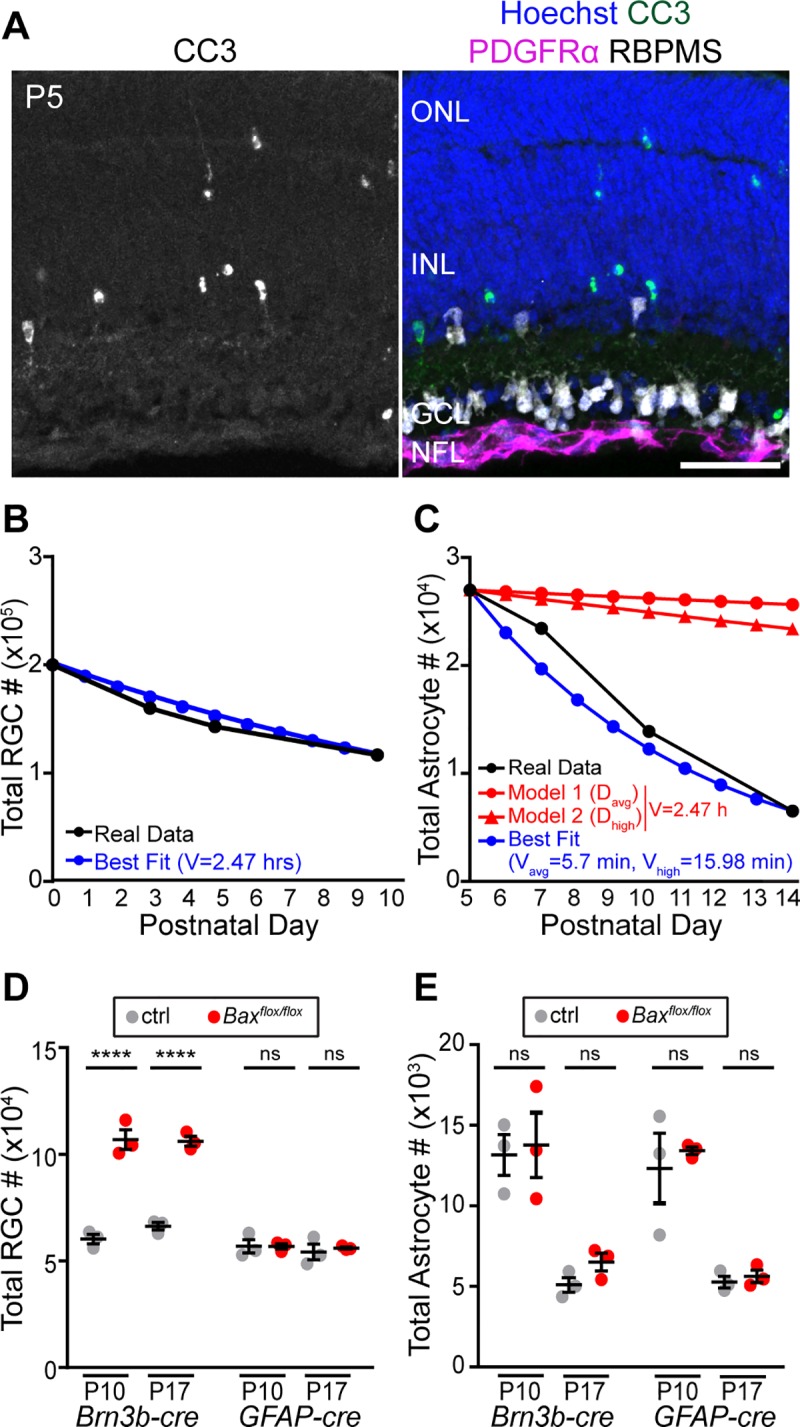
Retinal astrocyte loss is not due to apoptosis. (A) P5 retinal sections stained for CC3 (apoptosis marker), PDGFRα (astrocyte marker), RBPMS (RGC marker), and Hoechst (nuclear stain). CC3^+^ cells are frequently found in neuronal layers but not in the RNFL, where astrocytes reside. (B,C) Modeling to determine the effect of observed apoptosis rate on RGC (B) and astrocyte (C) population size. Black, observed cell number (B, RGC data from [35], see also S3D Fig; C, astrocyte numbers from Fig 1G). Blue, best fit models using observed apoptotic cell fraction as death rate parameter. Apoptotic RGC fraction (B) from [35] (see also S3D Fig). For astrocytes (C), both average (D_avg_) and maximum (D_high_) CC3^+^ astrocyte fractions from S3C Fig were used. Clearance time parameter V in the best fit model is given. Red in (C): astrocyte model with the clearance time parameter set to a biologically plausible value (2.47 hours); death rate set either to D_avg_ (circles) or D_high_ (triangles) (see S3C Fig). Apoptosis alone cannot account for the observed astrocyte numbers. (D,E) Effects of cell type–specific *Bax* deletion on RGC (D) and astrocyte (E) numbers. RGCs, *Brn3b-Cre*; astrocytes, *GFAP-Cre*. Statistics: for each cell type, three-way ANOVA followed by Tukey post hoc test (see S1 Table for ANOVA details); ****p < 0.0001. NS *p*-values ≥ 0.99. *N* = 3 for all groups. Error bars: mean ± SEM. Sample sizes depicted by data points on graph. For data plotted in graphs, see S1 Data. Scale bar, 50 μm. CC3, cleaved-caspase 3; ctrl, control; GCL, ganglion cell layer; INL, inner nuclear layer; NFL, nerve fiber layer; NS, not significant; ONL, outer nuclear layer; PDGFR, platelet-derived growth factor receptor; RGC, retinal ganglion cell; RNFL, retinal nerve fiber layer.

Because apoptosis is typically the mechanism for developmental cell death, we tested whether this was the case for retinal astrocytes. To this end, we stained retinas with antibodies to cleaved-caspase 3 (CC3), a histological marker of apoptotic cells [34]. Unexpectedly, few developing astrocytes expressed CC3 (Fig 2A; S3C Fig; *n* = 14 CC3^+^ astrocytes out of 29,383 analyzed across all ages). Therefore, astrocyte apoptosis was quite rare.

Apoptotic corpses can be cleared in a matter of hours, such that only a small fraction of the dying population is visible in any given histological sample [36,37]. To ask whether the small number of CC3^+^ astrocytes could reflect such a large change in cell number, we took a modeling approach. The model we used [37] was developed to estimate apoptotic cell clearance time, given experimental measurements of cell numbers and the fraction of visibly dying (i.e., CC3^+^) cells. If the clearance time is known, the model can also be used to calculate the expected cell number decline given a measurement of the CC3^+^ dying cell fraction. To validate this approach, we first modeled the developmental decline in RGC numbers previously reported in rat retina [35] (see Methods). The model’s best-fit curve predicted the RGC clearance time as 2.47 hours (Fig 2B), which is in alignment with published clearance times of approximately 1–3 hours for apoptotic neurons and oligodendrocytes [38–41]. We therefore applied the model to our astrocyte data. According to the model, in order for the observed percentage of apoptotic astrocytes to account for the observed cell loss, astrocyte clearance would need to occur in an implausibly short time—between approximately 5 and 15 minutes (Fig 2C, blue line). When the RGC clearance time was used (2.47 hours), the model predicted only a small decline in astrocyte number by P14, nowhere near the measured value (Fig 2C, red lines). These results strongly suggest that the observed frequency of astrocyte apoptosis is too low to plausibly account for the P5–P14 decline in cell number.

Given the scarcity of astrocyte apoptosis, we hypothesized that astrocyte death would proceed normally when apoptosis is genetically perturbed. *Bax* is an essential apoptotic gene, particularly in the CNS, where loss of its function impairs neuronal death and increases neuron numbers [14]. We crossed a conditional *Bax*^*flox*^ allele to a *GFAP-cre* line, which is highly selective for astrocytes and Müller glia [30] (S1B–S1D Fig). Deletion of *Bax* in astrocytes did not prevent astrocyte death (Fig 2E). By contrast, use of the same *Bax*^*flox*^ allele in combination with an RGC-specific Cre line increased RGC number approximately 2-fold, confirming previous reports [42,43] that deletion of *Bax* in RGCs rescues them from death (Fig 2D). These findings indicate that, unlike neurons, astrocytes die in a *Bax*-independent manner. Taken together, our CC3 and *Bax* studies demonstrate that apoptosis is not primarily responsible for astrocyte developmental death.

### Microglia engulf retinal astrocytes during their death period

**Fig 3:**
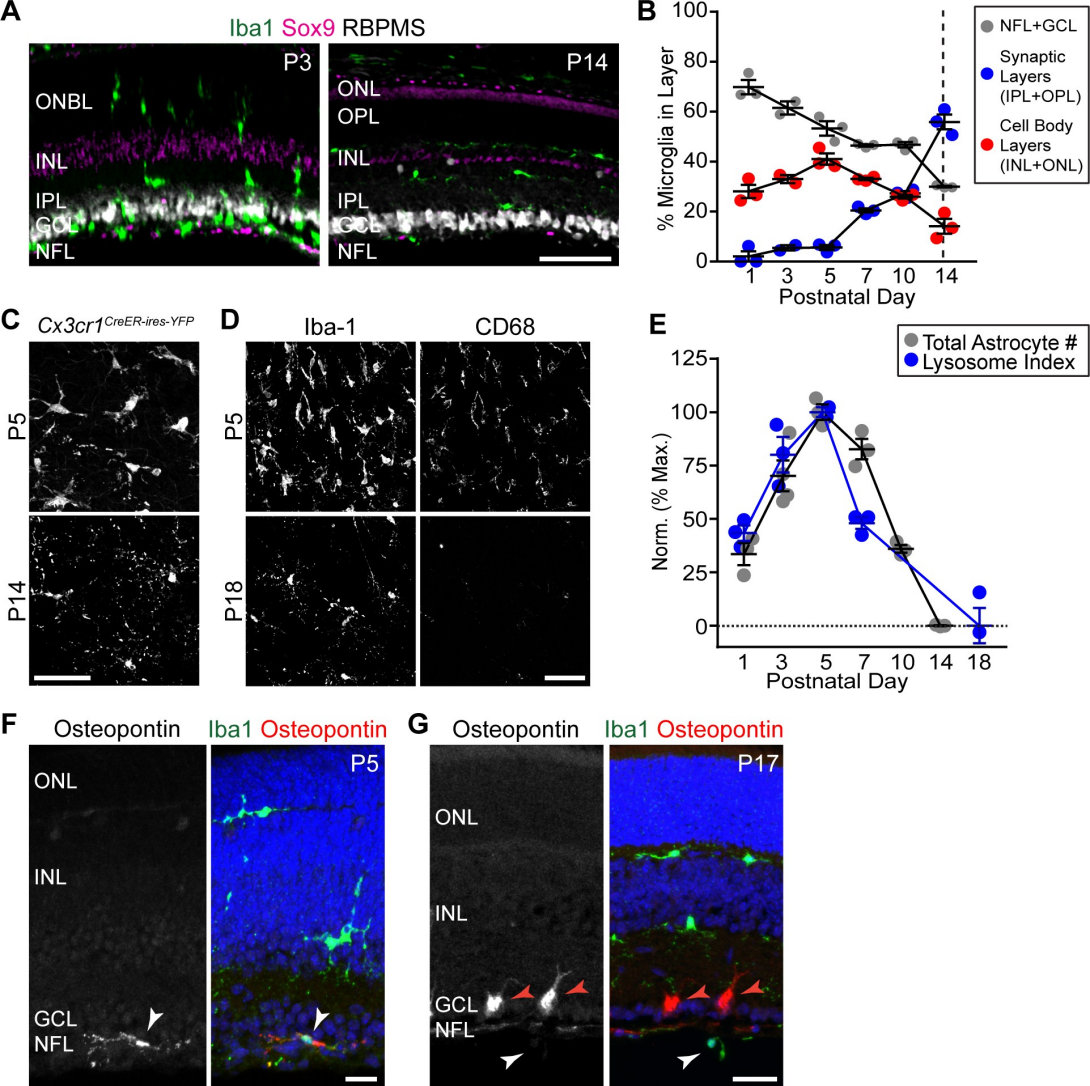
Nerve fiber layer microglia express a phagocytic phenotype during the astrocyte death period. (A,B) Retinal microglia localize preferentially to RNFL until the end of the astrocyte death period at P14 (dashed line). (A) Representative retinal sections showing laminar location of Iba-1^+^ microglia; (B) microglial laminar location quantified across development in whole-mount images (e.g., C). (C) RNFL microglia visualized in *Cx3cr1*^*CreER-ires-YFP*^ whole-mount using anti-GFP. Microglia show amoeboid morphology during the astrocyte death period (P5) but ramified morphology once death is complete (P14). (D) RNFL microglia (labeled with anti-Iba-1) have high lysosomal content (anti-CD68) at the peak of the astrocyte number (P5). CD68 is largely absent after the death period (P18). (E) Quantification of microglial phagocytic capacity, measured using a lysosome index (CD68^+^ lysosome content per microglial cell; blue). Gray, total astrocyte numbers, replotted from Fig 1G for comparison. Note the strong correlation between these measures, both during arrival of astrocytes in retina (P1–P5; see Fig 1A) and during their elimination (P5–P14). (F,G) During the death period, RNFL microglia (white arrows) are molecularly distinct from Iba-1^+^ microglia in other layers, as shown by their expression of Osteopontin at P5 (F) but not P17 (G). Red arrows, Osteopontin^+^ RGCs [48]. Error bars, mean ± SEM. Sample sizes are depicted by data points on graph. For data plotted in graphs, see S1 Data. Scale bars, 50 μm (A,C,D); 20 μm (F,G). GCL, ganglion cell layer; GFP, green fluorescent protein; INL, inner nuclear layer; IPL, inner plexiform layer; NFL, nerve fiber layer; ONBL, outer neuroblast layer; ONL, outer nuclear layer; OPL, outer plexiform layer; RGC, retinal ganglion cell; RNFL, retinal nerve fiber layer.

**Fig 4:**
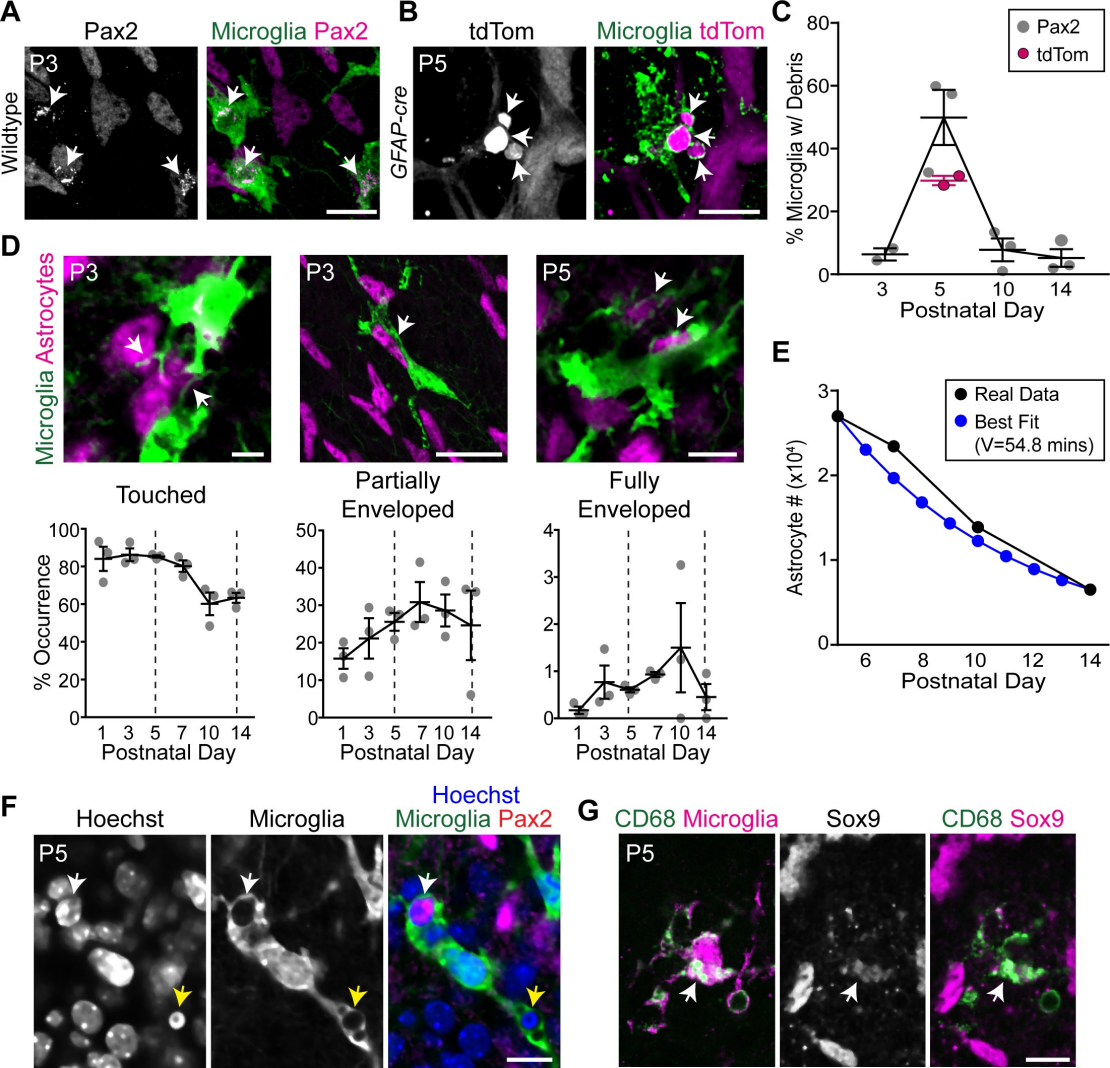
Microglia engulf developing retinal astrocytes. (A,B) Representative images showing astrocyte debris within microglia (arrows). Astrocytes labeled with antibody against Pax2 (A) or with tdTomato (tdTom) Cre reporter driven by *GFAP-cre* (B). This Cre line is selective for astrocytes at P5 and is not yet expressed by Müller glia [30]. Microglia labeled with anti-GFP in *Cx3cr1*^*CreER-ires-YFP*^ mice (A) or antibodies to Iba-1 and P2Y12 (B). (A) single optical plane; (B) Z-projection of three optical planes (1.2 μm Z-distance total). Also see S4 Fig for 3D reconstructions of astrocyte debris within microglia. (C) Percentage of microglia containing astrocyte debris peaks at P5, coinciding with peak astrocyte number. (D) Astrocyte–microglia interactions quantified in *Cx3cr1*^*CreER-ires-YFP*^ mice. Bottom row, percentage astrocyte somata touched, partially enveloped, or fully enveloped by microglial processes. Examples of each interaction category are shown (top row; arrows). Dashed lines: start (P5) and end (P14) of astrocyte loss. Phagocytosis-like interactions (i.e., partially or fully enveloped astrocyte somata) peak during the death period. Also see S1 Movie, S2 Movie and S3 Movie for 3D reconstructions of microglia–astrocyte interactions. (E) Modeling suggests that engulfment rate is sufficient to account for developmental changes in astrocyte population size. Black, observed astrocyte number from Fig 1G. Blue, best-fit model using average frequency of engulfment determined in (D) (“fully enclosed” category) as death rate parameter. The clearance time parameter of best fit model (54.8 minutes) is plausible given past studies [18,49]. (F) Engulfed astrocytes do not have pyknotic nuclei. White arrow, non-pyknotic astrocyte nucleus within a microglial phagocytic cup. Yellow arrow, non-astrocytic pyknotic nuclei within phagocytic cup of the same microglial cell. A single optical plane is shown. Microglia are labeled with *Cx3cr1*^*CreER-ires-YFP*^. (G) Astrocyte debris (arrow) within CD68^+^ lysosomal compartments of *Cx3cr1*^*CreER-ires-YFP*^ microglia. All images are en face views of whole-mounted retina. Error bars, mean ± SEM. Sample sizes are depicted by data points on graphs. For data plotted in graphs, see S1 Data. Scale bars, 5 μm (D, left); 10 μm (D, right; F; G); 25 μm (A; B; D, center). GFP, green fluorescent protein; tdTom, tandem dimer Tomato; YFP, yellow fluorescent protein.

We next investigated non-apoptotic death programs that might eliminate astrocytes. Electron micrographs of the developing RNFL showed no histological hallmarks of autophagy or necroptosis [34], arguing against involvement of these mechanisms (S3B Fig). To explore a role for microglia, we began by examining their localization and morphology, using *Cx3cr1*^*CreER-ires-YFP*^ mice [44] (abbreviated *Cx3cr1*^*CreER*^). Microglia preferentially accumulated in the RNFL during the astrocyte death period (Fig 3A and 3B) and sent out processes contacting the vast majority of astrocytes (Fig 4D). Furthermore, during the death period, RNFL microglia exhibited both morphological and molecular signatures of phagocytic activity. Morphologically, they had an amoeboid shape typical of phagocytic macrophages [45] (Fig 3C). Molecularly, they selectively expressed CD68 and Osteopontin, two markers of phagocytic microglia [46,47] (Fig 3D–3F). Once the death period was over, however, microglia assumed a resting morphology and down-regulated both markers (Fig 3C–3G). Together, these observations demonstrate that phagocytic microglia are present at the right time and place to play a role in developmental astrocyte death.

To test whether the phagocytic state of RNFL microglia reflected engulfment of astrocytes, we analyzed retinal tissue double-labeled for each cell type. Astrocyte nuclear debris, immunopositive for Pax2 or Sox9, was found within microglial intracellular compartments (Fig 4A, 4C and 4G; S4A Fig; S1 Movie). These compartments co-labeled for CD68, consistent with the expected lysosomal destination of engulfed material (Fig 4G). Internalization of astrocyte debris was also confirmed using tdTomato as a genetically encoded astrocyte label (Fig 4B and 4C; S4B Fig). Furthermore, we observed microglial processes that partially or fully enveloped astrocyte somata, suggesting they were being engulfed (Fig 4D; S2 Movie; S3 Movie). These processes often resembled the classic phagocytic cups that surrounded pyknotic neuronal corpses (Fig 4F). Notably, the nuclei of engulfed astrocytes were not pyknotic or degenerated (Fig 4F), suggesting they may be engulfed while still viable. Engulfment was detected at all sampled locations across the center-peripheral axis, at all ages between P1 and P14. Therefore, given the time line of astrocyte maturation (Fig 1H) [33], both immature precursors and mature astrocytes were subject to phagocytosis. Anatomical signatures of astrocyte engulfment were detected most frequently during the period of astrocyte death, suggesting that engulfment occurred selectively during this time (Fig 4C and 4D). Therefore, the phagocytic state of RNFL microglia during the death period is due at least in part to their engulfment of astrocytes.

To ask whether engulfment occurs often enough to explain the decline in astrocyte numbers, we once again took a modeling approach. Using the same model as in our apoptosis studies (Fig 2C), we calculated astrocyte clearance time based on the observed average number of “fully enveloped” astrocytes (Fig 4D). The model (Fig 4E) predicted a clearance time of 54.8 minutes, closely matching previous reports of the time needed for microglia to clear engulfed cells (approximately 45 minutes) [18,49]. Therefore, astrocyte engulfment occurs with sufficient frequency to plausibly account for astrocyte death.

### Blockade of major phagocytosis pathways does not impact astrocyte number

**Fig 5:**
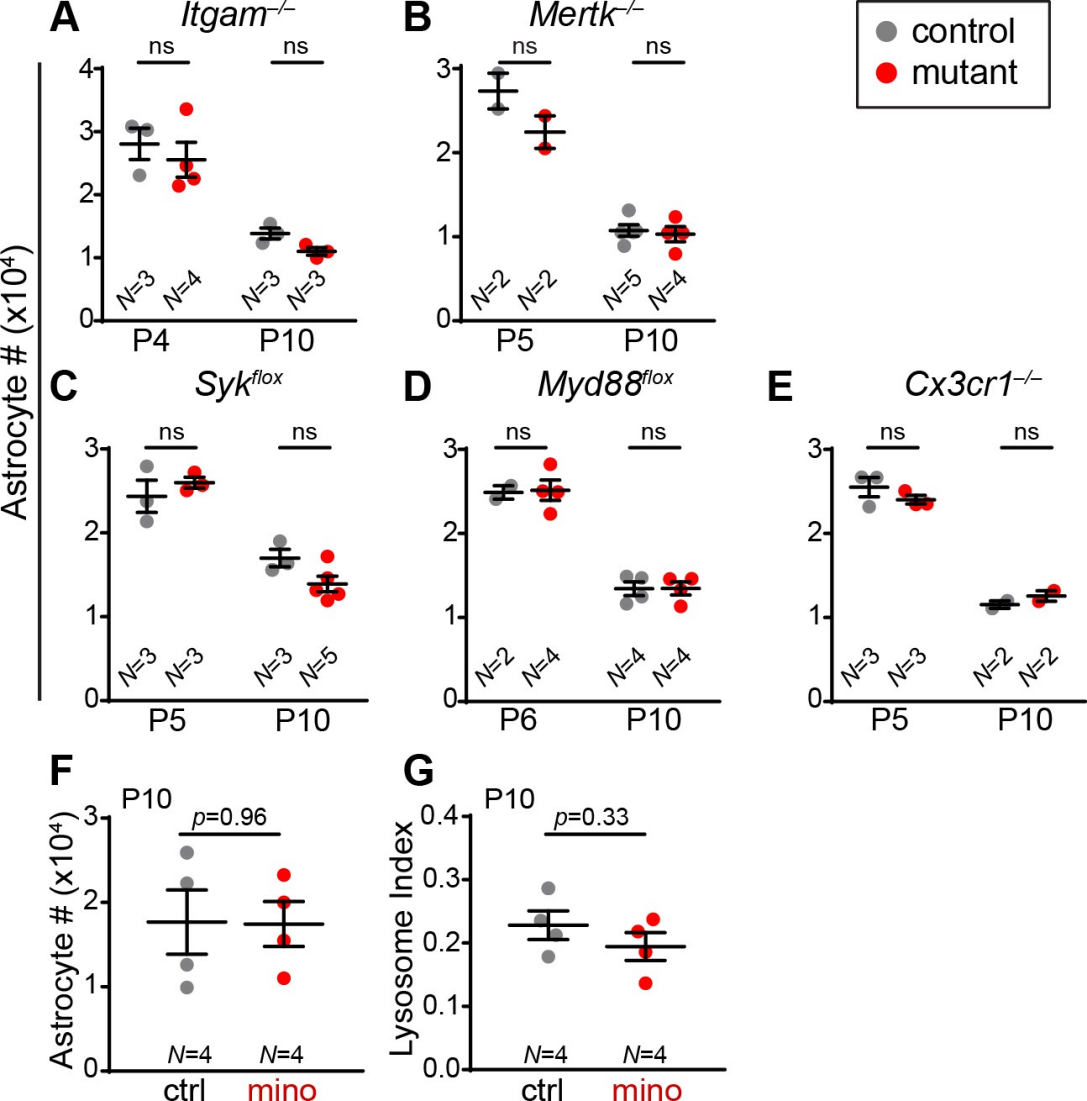
Blockade of major phagocytosis pathways does not affect astrocyte number. (A-E) Total astrocyte numbers were determined in mutant mice (red) and littermate controls (gray) at two ages during the astrocyte death period. (A) *Itgam* constitutive null mutants lack the complement C3 receptor. (B) *Mertk* constitutive null mutants lack Mer tyrosine kinase receptor. (C,D) *Syk*^*flox*^ (C) and *Myd88*^*flox*^ (D) are conditional alleles of signaling components downstream of FcγR/TREM2 (C) or Toll-like receptors (D). Mutants carried *Cx3cr1*^*CreER*^ (C) or *Cx3cr1-Cre* (D) to drive microglia-specific gene deletion. (E) *Cx3cr1*^*CreER/CreER*^ constitutive null mice lack fractalkine receptor. Statistics: for each data set, two-way ANOVA was performed; none resulted in significant main effects of genotype on astrocyte number (see S1 Table for ANOVA details). (F,G) No effect of minocycline on total astrocyte numbers (F) or microglial phagocytic capacity (G). Administration, P4–P9; analysis, P10. Statistics: two-tailed *t* tests. Error bars, mean ± SEM. For data plotted in graphs, see S1 Data. ctrl, control; mino, minocycline; ns, not significant.

The results so far led us to hypothesize that microglial phagocytosis is responsible for causing astrocyte death. To test this possibility, we first sought to prevent microglia from engaging in phagocytosis. Microglia express several well-characterized receptors that are required for engulfment of dead cells, cellular debris, and in some cases even viable cells [16,50–54]. We reasoned that one of these pathways might also mediate elimination of developing astrocytes. We therefore examined mutant mice in which the best characterized receptors or their downstream signaling components were eliminated. These included mutants lacking the complement C3 receptor CR3 (*Itgam*^*–/–*^); the Mer tyrosine kinase receptor (*Mertk*^*–/–*^); and Syk tyrosine kinase (*Cx3cr1*^*CreER*^; *Syk*^*flox/flox*^*)*, an essential signal downstream of FC receptors and TREM-2 [53,55]. If these pathways are required for astrocyte elimination, astrocyte numbers should be increased in mutants. However, none of the mutants showed such an effect (Fig 5A–5C).

We next investigated the role of two well-characterized signaling pathways that are potent regulators of microglial physiology, including their phagocytic activity. First, we tested the role of the pro-inflammatory Toll-like receptors using a microglia-specific deletion of their essential downstream signaling molecule MyD88 (*Cx3cr1-Cre; Myd88*^*flox/flox*^). Second, we examined mice lacking the CX3CR1 receptor (*Cx3cr1*^*CreER/CreER*^). Neither mutant showed an astrocyte survival phenotype (Fig 5D and 5E). Finally, we tested minocycline, a drug that suppresses inflammation and microglial phagocytic activity through an unknown mechanism. This too failed to affect microglial phagocytic activity, as measured by both CD68^+^ lysosome content and astrocyte numbers (Fig 5F and 5G). Taken together, these experiments indicate that the best-characterized pro-inflammatory and pro-engulfment microglial pathways are either not involved in astrocyte death, or they are capable of completely compensating for each other when one is absent.

### Ablation of microglia increases astrocyte number

One possible interpretation of the receptor mutant studies is that microglia mediate astrocyte removal through a new pathway or through a combination of the tested pathways. However, it also remains possible that microglia are not in fact involved in astrocyte developmental death. To distinguish between these possibilities, we ablated microglia during the death period, starting at P4 and continuing until the end of the death period at P14. If microglia are responsible for culling the astrocyte population, then ablating microglia during this period should prevent astrocyte death.

**Fig 6:**
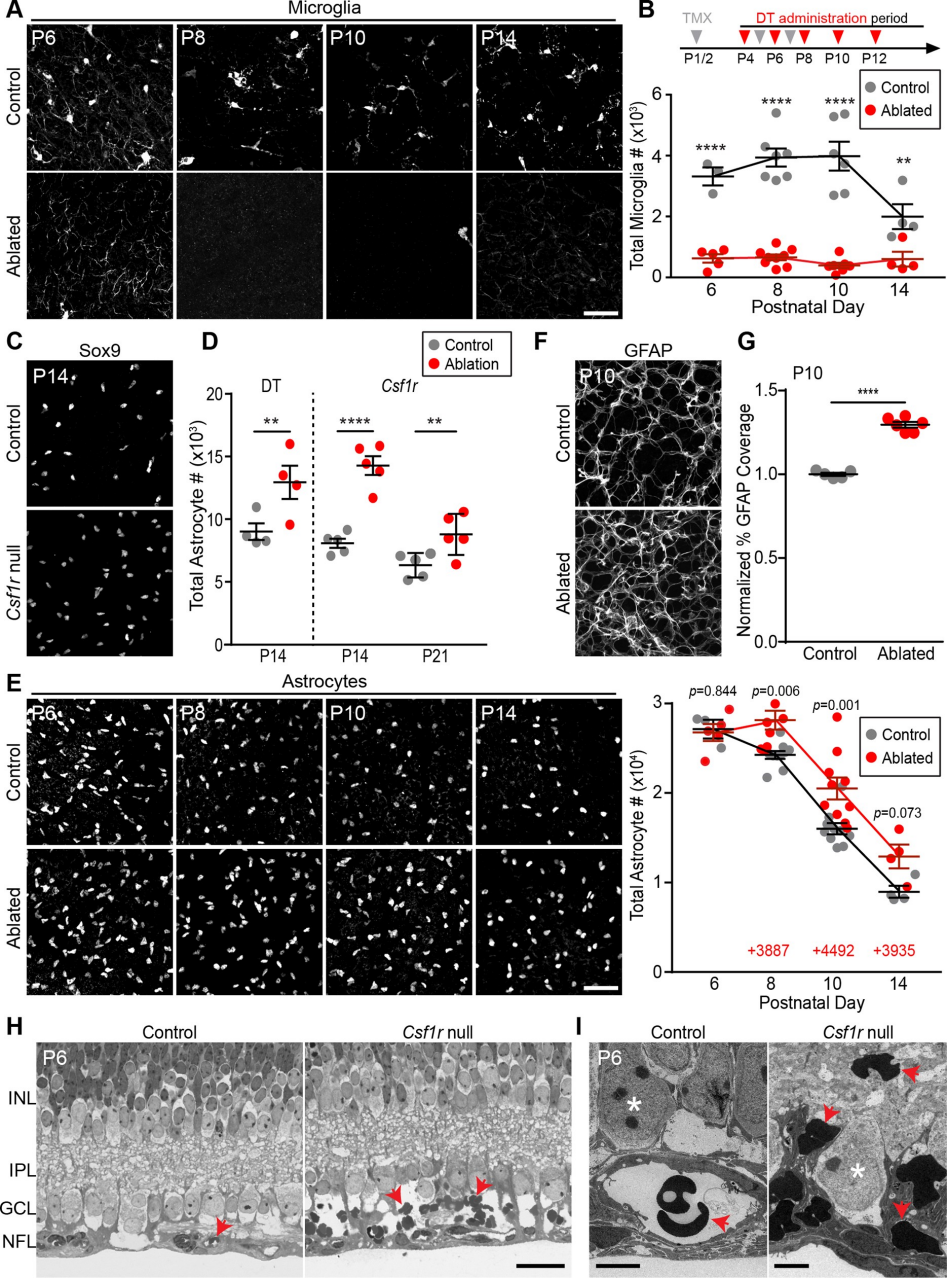
Ablation of microglia increases astrocyte number. (A,B) Microglial ablation paradigm. Tamoxifen (TMX, gray arrows) and diphtheria toxin (DT, red arrows) were administered at indicated times (B, top). (A) Representative confocal images of whole-mount *Cx3cr1*^*CreER-ires-YFP*^ retina showing microglial morphology (anti-GFP). (B) Quantification of microglial numbers. Ablated mice carried both *Cx3cr1*^*CreER*^ and *Rosa26*^*iDTR*^ transgenes; littermate controls lacked one of the transgenes. Statistics: two-way ANOVA followed by post hoc tests specified in S1 Table. ****p < 0.0001; **p = 0.0037. (**C,D**) Elimination of microglia using DTR paradigm or *Csf1r* null mice increases astrocyte number at P14. The extent of increase was similar in each paradigm. Some additional astrocyte loss occurs after the usual death period, but numbers remain higher at P21. Statistics: two-way ANOVA followed by post hoc tests specified in S1 Table. **p = 0.01; ****p < 0.0001. (**E**) Effects of DTR microglial ablation on astrocyte number across development. Left, representative images of Pax2^+^ or Sox9^+^ astrocytes in control and microglia-ablated animals. Right, quantification of astrocyte number. Red text, number of excess astrocytes in ablated animals at each age. Statistics: two-way ANOVA followed by post hoc tests specified in S1 Table. (**F,G**) Increased density of GFAP^+^ astrocyte network in ablated retina. (F) Representative en face confocal images of RNFL. (G) Quantification of area covered by GFAP^+^ arbors, from images similar to F. Statistics: two-tailed *t* test (*p* < 0.0001). (**H,I**) Thin plastic sections of P6 *Csf1r* null mutant retina and littermate control, viewed by light microscopy (I) or electron microscopy (J). In controls, red blood cells (arrows) are located within vascular lumen. In mutants, extravascular red blood cells accumulate in the NFL-GCL region. Asterisks in J, RGC somata. The same phenotype was observed at P14; see S7D Fig. Error bars, mean ± SEM. Sample sizes are denoted by data points on graphs. For data plotted in graphs, see S1 Data. Scale bars, 50 μm (A,C,E,F); 25 μm (H); 5 μm (I). DT, diphtheria toxin; DTR, diphtheria toxin receptor; GCL, ganglion cell layer; GFAP, glial fibrillary acidic protein; GFP, green fluorescent protein; INL, inner nuclear layer; IPL, inner plexiform layer; NFL, nerve fiber layer; RGC, retinal ganglion cell; RNFL, retinal nerve fiber layer; TMX, tamoxifen.

To ablate microglia, we used an established chemogenetic strategy [44] whereby *Cx3cr1*^*CreER*^ drives microglia-specific expression of a tamoxifen-inducible diphtheria toxin receptor (DTR). Because these tools had not previously been used during retinal development, we first confirmed their specificity, efficacy, and temporal characteristics (S5 and S6 Figs). Based on these experiments, we developed a protocol for repeated tamoxifen and diphtheria toxin administration that selectively eliminated microglia as early as P6 and prevented their return for the entirety of the death period (Fig 6A and 6B). When microglia were ablated in this manner, the number of astrocytes remaining at P14 was increased relative to littermate controls. The same was true when we eliminated microglia in an entirely different way, using a constitutive null *Csf1r* mutant mouse strain [56] (Fig 6C and 6D). Therefore, absence of microglia increases astrocyte number.

To determine how this increase arises, we generated a developmental time course of astrocyte numbers in our DTR microglia ablation paradigm. Astrocyte numbers failed to decline between P6 and P8, leading to an excess of approximately 4,000 astrocytes relative to their littermate controls (Fig 6E). This excess was not caused by increased proliferation (S6E and S6F Fig), indicating that it was instead due to impaired death. After P8, astrocyte numbers began declining again in microglia-ablated animals, suggesting the existence of compensatory death mechanisms (see below for further investigation of this phenomenon). Despite this compensation, astrocyte numbers failed to return to normal levels by the end of the death period. This was evident in both DTR and *Csf1r* mutant paradigms (Fig 6D and 6E; S6C Fig). In fact, even a week after the normal end of the death period, astrocyte numbers were still elevated (Fig 6D).

To ensure that this result could be validated with other microglial Cre lines, we also used a second transgenic mouse, *Cx3cr1-Cre* [57], to drive DTR expression. Retinal microglia were also effectively ablated in this strain, and effects on astrocyte number were similar in magnitude, regardless of which Cre line was used (Fig 6C and 6D; S6D Fig). Together, these findings support the conclusion that microglia depletion impairs the developmental loss of astrocytes.

### Astrocytes that are spared in microglia-deficient mice survive and differentiate

The preservation of cells expressing astrocyte nuclear markers (Fig 6C–6E) could mean that microglia are required for astrocyte death; alternatively, this could mean that microglia are required merely for the clearance of dead or dying astrocytes, similar to their role in apoptosis [36]. In the former case, we would expect that the excess astrocytes in microglia-ablated mice should become mature, both molecularly and anatomically. However, if microglia mediate clearance but not death, ablation would be expected to increase the number of degenerating corpses without a similar increase in the number of differentiated astrocytes. To distinguish between these possibilities, we performed two histological analyses. First, we used electron microscopy to investigate the morphology of astrocytes in microglia-deficient *Csf1r*^*–/–*^mice (*n* > 100 astrocytes; *N* = 4 mutants). No degenerative changes in astrocyte morphology were evident; indeed, astrocytes were virtually indistinguishable from those in control mice (S7D Fig; compare with S3B Fig).

**Fig 7:**
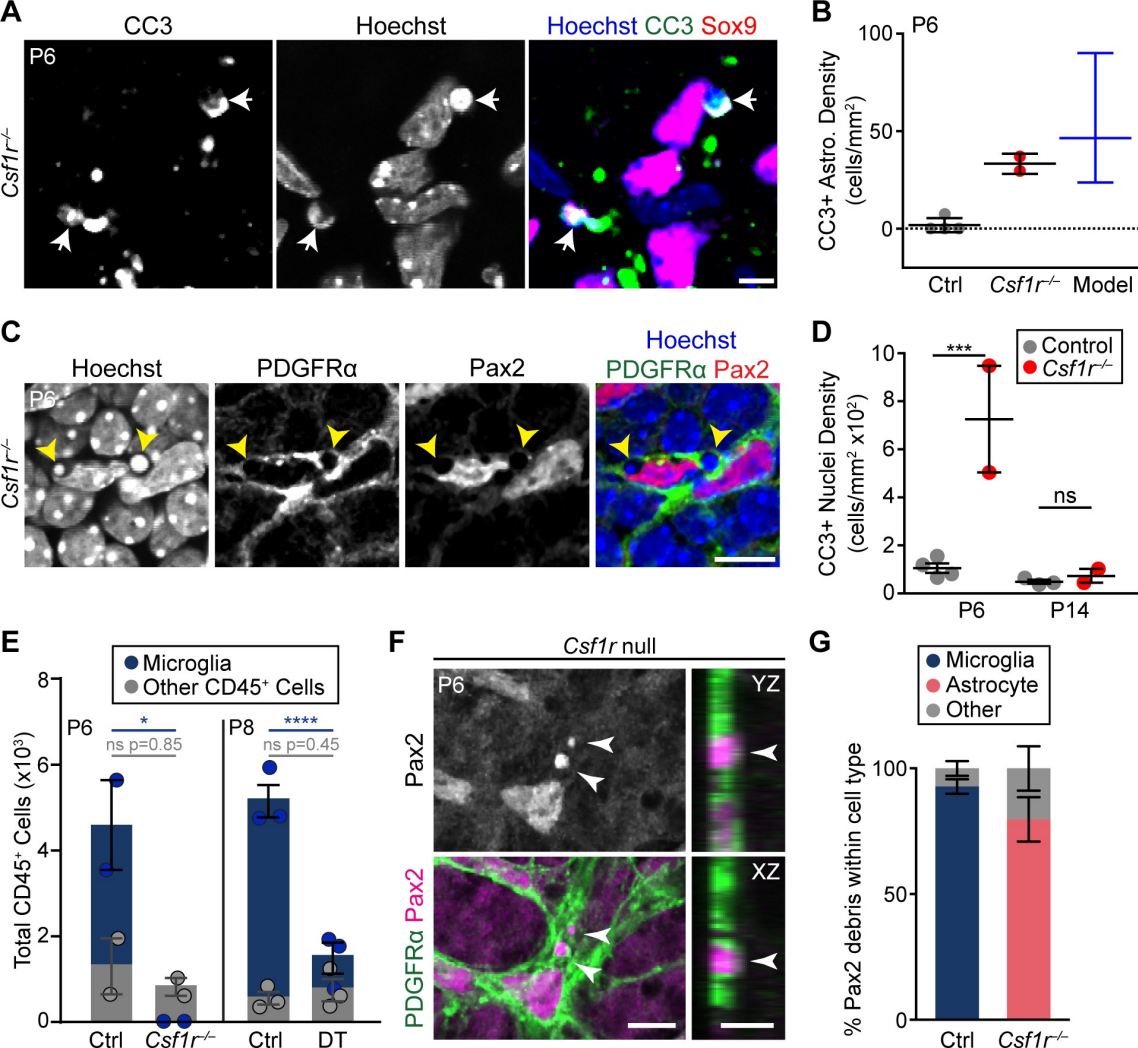
Compensatory mechanisms for astrocyte death in the absence of microglia. (A,B) Example images (A) and quantification (B) of CC3^+^ astrocytes (arrows) in *Csf1r*^–/–^retina. Model (B, blue) shows expected density of CC3^+^ astrocytes under a scenario in which apoptosis occurs at a wild-type rate but no corpses are cleared (see Methods). The observed value from *Csf1r* null mutants (C, red) falls within the 95% confidence interval of the model. (C,D) Non-professional phagocytes clear apoptotic corpses when microglia are absent. (C) En face view of *Csf1r*^*–/–*^retinal whole-mount (single optical plane) showing pyknotic bodies (arrows, Hoechst channel) surrounded by PDGFRα^+^ astrocyte processes resembling phagocytic cups. Such features were never observed in wild-type mice. (D) Delayed clearance of apoptotic corpses occurs between P6 and P14 in *Csf1r* mutants. All CC3^+^ nuclei within the NFL and GCL were quantified at P6 and P14. These are largely apoptotic neurons or endothelial cells; few are astrocytes (B). Statistics: two-way ANOVA followed by post hoc tests specified in S1 Table. P6, ***p = 0.0006; P14, *p* = 0.8340. (E) Quantification of CD45^+^ cells in control and microglia-ablated retina. The non-microglial fraction of CD45^+^ cells (gray) was unchanged following acute (DT) or chronic (*Csf1r*) microglial ablation. Statistics: two-way ANOVA followed by post hoc tests specified in S1 Table; *p = 0.0400; ****p < 0.0001. (F) Left: en face view (single optical plane) of RNFL in whole-mount *Csf1r* mutant retina. Pax2^+^ astrocyte debris localizes within PDGFRα^+^ astrocyte processes (arrows). Right: orthogonal YZ and XZ views generated from Z-stack. Pax2^+^ debris is encapsulated by PDGFRα^+^ processes. (G) Quantification of the percentage of Pax2^+^ debris localized to microglia, astrocytes, or other cell types in control mice and in *Csf1r* mutants (P5–P6). Most Pax2^+^ debris is found within astrocytes when microglia are absent. Error bars, mean ± SEM (except [C]; blue, mean ± 95% confidence interval). Sample sizes are denoted by data points on graphs. For data plotted in graphs, see S1 Data. Scale bars, 5 μm (A, F right panels); 10 μm (C, F left panel). CC3, cleaved-caspase 3; Ctrl, control. DT, diphtheria toxin; GCL, ganglion cell layer; NFL, nerve fiber layer; ns, not significant; PDGFR, platelet-derived growth factor receptor; RNFL, retinal nerve fiber layer.

Second, to investigate molecular and morphological differentiation, we stained microglia-ablated retinas for GFAP, a well-validated retinal astrocyte marker that is expressed at a late stage of their differentiation [28,30,33,58] (Fig 1H). Nearly all (>98.8%) Pax2^+^ or Sox9^+^ astrocytes co-expressed GFAP by P10, regardless of whether microglia had been ablated (Control, *n* = 2,642 astrocytes; DTR, *n* = 4,145 astrocytes; *N* = 3 animals per condition). Thus, molecular differentiation of excess astrocytes was normal. Excess astrocytes also incorporated normally into the orderly mosaic of astrocyte cell bodies [30,59], suggesting that they engaged in normal repulsive interactions with their homotypic neighbors (S7A and S7B Fig). Furthermore, the GFAP^+^ arbor network covered significantly more area in animals lacking microglia (Figs 6F and 7G); the magnitude increase was similar to the increase in total astrocyte number (P10: 30% arbor area increase over littermate controls; 28% cell number increase). This finding suggests that excess astrocytes undergo morphological differentiation. Together these results support the view that spared astrocytes are not corpses but rather have the capacity to develop normally.

### Retinal hemorrhage in the absence of microglia-mediated phagocytosis

We next investigated the functional role of microglia-mediated astrocyte death in retinal histogenesis. It has previously been shown that an excess of astrocytes leads to retinal hemorrhage, likely due to the close developmental association between astrocytes and vasculature [22,32]. We therefore hypothesized that a similar vascular phenotype should be evident in *Csf1r* mutant mice, which also have excess astrocytes (Fig 6D). In *Csf1r* mutants, overall vascular patterning was largely normal, aside from some early subtle changes consistent with those previously reported for mice lacking microglia [60] (S7F Fig). However, histological examination of *Csf1r* mutant retina at P6 and P14 revealed bleeding within the RNFL. In thin plastic sections from littermate controls (P6, Fig 6H and 6I, *N* = 3; P14, S7E Fig, *N* = 4), red blood cells (RBCs) were confined to the vasculature. By contrast, in mutants, numerous RBC profiles were observed throughout the RNFL extracellular space (Fig 6H and 6I; S7D and S7E Fig; *N* = 4 at each age, all of which were affected). Thus, microglia are required for vessel integrity during retinal angiogenesis. This finding is consistent with a model in which the microglial requirement for vascular integrity is mediated indirectly through microglial effects on astrocyte number.

### Compensatory mechanisms for astrocyte death in the absence of microglia

Ablation of microglia did not completely block astrocyte death; instead, death appeared to stop for approximately 2 days before resuming again at virtually the same rate as in controls (Fig 6E). A similar phenomenon was observed in *Csf1r* mutants: as astrocytes migrated centrifugally (Fig 1A), their arrival in a given retinal region was followed by an approximate 2-day period when astrocyte loss was impaired (S8A Fig). Subsequently, loss of astrocytes resumed with similar dynamics as controls, albeit with the decline curve shifted rightwards (S8A and S8B Fig). These observations suggest that accumulation of excess astrocytes triggers compensatory death when microglia are absent.

To investigate the nature of the compensatory mechanisms, we used *Csf1r* mutants to examine three possible alternate death routes. First, we tested whether astrocyte apoptosis might become more frequent in the absence of microglia. At P6, the number of CC3^+^ astrocytes was elevated in *Csf1r* mutants (Fig 7A and 7B); however, this increase does not necessarily imply an increased apoptosis rate, because microglia are required for clearance of apoptotic corpses [51] (Fig 7D). Therefore, even if astrocytes undergo apoptosis at a wild-type rate (S3C Fig), the failure of clearance would be expected to increase the number of detectable CC3^+^ corpses. To ask whether changes in apoptosis rate contribute to the CC3 phenotype, we performed a modeling study. If the CC3 phenotype is due solely to clearance defects, it should be possible to use the measured wild-type apoptosis rate (S3C Fig) to predict the number of uncleared CC3^+^ astrocytes remaining in mutants. By contrast, if the apoptosis rate has increased in mutants, a prediction based on the wild-type apoptosis rate should underestimate how many CC3^+^ astrocytes are found in mutants. Consistent with the first of these two possibilities, we found that the predicted and actual numbers of mutant CC3^+^ astrocytes were quite similar (Fig 7B). This finding indicates that the apoptosis rate has not changed in the absence of microglia. Further supporting this conclusion, CC3 staining in our acute DTR ablation model also failed to detect elevated astrocyte apoptosis rates (S8D Fig). Thus, apoptosis is unlikely to be the compensatory astrocyte death mechanism.

Next, we tested whether loss of microglia causes another immune cell type to assume the role of phagocyte. *Csf1r*^*–/–*^and littermate control retinas were stained with antibodies to CD45, a broad leukocyte marker. In controls, most retinal CD45^+^ cells were microglia, but a small population of non-microglial CD45^+^ cells (<2,000 per retina) was present during the period of astrocyte death (Fig 7E; S8E Fig). When microglia were eliminated using either *Csf1r* mutants or the toxin ablation model, the size of the remaining CD45^+^ population was similar to controls, arguing against any major infiltration of leukocytes (Fig 7E; S8E Fig). Moreover, it is unlikely that the resident CD45^+^ cells were able to compensate on their own for the loss of microglia because we never observed any astrocyte material within these cells. For these reasons, leukocytes are poor candidates to mediate compensatory astrocyte elimination.

### Developing astrocytes engulf each other in the absence of microglia

**Fig 8:**
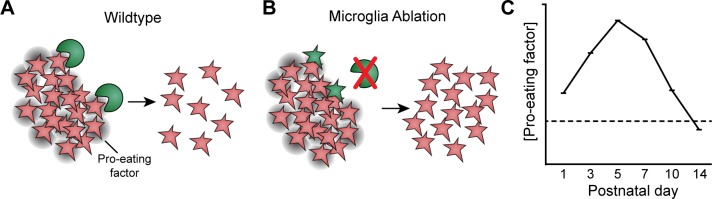
Model of the mechanisms driving developmental astrocyte death. Schematic illustrating the proposed mechanism of developmental astrocyte death, based on findings of this study. In wild-type mice (**A**), microglia (green) sense the number of astrocytes (red) within their local environment, perhaps due to expression of an astrocyte-derived pro-eating factor (gray shading; right panel). When astrocyte numbers are high, microglia are stimulated to remove them via phagocytosis. As astrocyte number falls, the amount of astrocyte-derived cue eventually becomes insufficient to stimulate microglia, thereby closing the death period (**C**). When microglia are ablated (**B**), death is temporarily halted. But astrocytes (green) eventually sense their own elevated numbers (red), leading them to phagocytose each other.

Finally, we investigated a third possible route of compensatory astrocyte death: We asked whether microglia depletion might cause another retinal cell type to assume “death by phagocyte” functions. This is plausible because, in other contexts, non-professional phagocytes are able to take on engulfment responsibilities when professional phagocytes are absent [61,62]. Non-professional phagocytes were likely active in *Csf1r* mutant retina, because CC3^+^ apoptotic corpses were eventually removed following an initial clearance defect (Fig 7D; S8D Fig). At least some of this compensatory corpse clearance was performed by RNFL astrocytes (Fig 7C; S9A Fig), raising the possibility that astrocytes might also take on other microglial phagocytic functions. Furthermore, ultrastructural analysis of astrocytes in microglia-deficient retinas revealed electron-dense structures resembling phagosomes [63] (S7D Fig). These structures were never observed in wild-type astrocytes (S3B Fig), suggesting that astrocytes increase their phagocytic capacity in the absence of microglia. We therefore tested whether absence of microglia might cause astrocytes to phagocytose each other. Using the Pax2 debris assay (Fig 4A and 4C), we found that dead astrocyte material was readily identifiable in *Csf1r* mutants (Fig 7F; S9B Fig). Unlike controls, in which Pax2 debris localized almost exclusively to microglia, debris in *Csf1r*^*–/–*^retina was largely found within PDGFRα^+^ astrocytes (Fig 7F and 7G; *n* = 564 debris puncta from 3 controls, 93% ± 2.9% in microglia; *n* = 111 debris puncta from 3 mutants; 80% ± 8.9% in astrocytes). This finding suggests that astrocytes themselves are the major cellular effector of astrocyte engulfment when microglia are absent. Our results are consistent with a model whereby astrocytes compensate for microglial ablation by killing and engulfing each other (Fig 8).

## Discussion

It has generally been assumed that developmental astrocyte death proceeds in a manner similar to neurons and oligodendrocytes, both of which die by apoptosis in large numbers [4,39]. However, these assumptions have rarely been tested in a quantitative manner; as a result, it remains unclear whether astrocytes are even subject to large-scale developmental death, let alone whether apoptosis is the mechanism. Here, we demonstrate that retinal astrocytes undergo developmental death that is not apoptotic but instead proceeds by a microglial “death by phagocyte” mechanism. The number of astrocytes killed by microglia is remarkably large: it includes not only the >3-fold cell number decline between P5 and P14, but also a fraction of astrocytes that are consumed during their migratory period, prior to P5 (Fig 3E and Fig 4). Microglia are necessary for normal progression of astrocyte death, because death was reduced upon elimination of microglia from developing retina. Furthermore, we show that in the absence of microglia, astrocytes themselves become phagocytic and engulf their homotypic neighbors. This activity appears to partially compensate for the loss of microglia in mediating astrocyte removal. Nevertheless, elimination of microglia halts death for approximately 2 days, long enough to produce an increase in the number of viable, differentiated astrocytes that persists until at least P21. This increase in astrocyte number may have functional consequences, because mice lacking microglia exhibit bleeding from the astrocyte-associated RNFL vasculature. Overall, these findings suggest that microglia-mediated death impacts the anatomy of the RNFL astrocyte network, and they raise the possibility that similar mechanisms may operate elsewhere in the CNS.

### Death by phagocyte as an astrocyte death mechanism

Five key findings support our conclusion that microglia are responsible for developmental astrocyte death. First, microglia in the RNFL assume a distinctive phagocytic phenotype and consume astrocyte debris. Second, these behaviors are limited to the period when astrocytes are dying. Third, the frequency of observed astrocyte engulfment events is sufficient to explain the decline in astrocyte number. Fourth, experimental elimination of microglia reduces astrocyte loss. Fifth, the astrocytes preserved in microglial ablation experiments go on to differentiate, suggesting they have been spared from death. For these reasons, we conclude that microglia kill retinal astrocytes. The phagocyte behavior we describe is fundamentally different from the well-described phenomenon of apoptotic corpse clearance, because interfering with phagocyte function does not typically rescue apoptotic cells from death [36] (S8C Fig). Astrocyte killing may be a case of “phagoptosis”—a form of cell death that can be prevented by blocking phagocytosis [14]. Because astrocytes may be engulfed while still viable (Fig 4F), we favor the notion that phagocytosis is in fact the death-inducing event. However, because we have yet to identify a phagocytosis pathway required for death, our data do not rule out a scenario in which microglia first kill astrocytes via a separate (non-apoptotic) pathway before immediately engulfing the doomed cells. To accommodate this possibility, we have avoided the term phagoptosis and prefer the term death by phagocyte.

There is precedent for the notion that microglia can mediate death by phagocyte, both in pathological contexts [15–17] and during development of cortical subventricular zone progenitors [18]. Furthermore, a recent study showed that retinal microglia can engulf embryonic RGCs [64], adding to the plausibility of our conclusions. One important difference between astrocytes and these other two cell types is that astrocytes rarely undergo apoptosis (Fig 2). By contrast, RGCs and cortical progenitors are both subject to significant apoptotic death [40,43], suggesting that death by phagocyte may play more of a complementary role. Indeed, our results in *Bax* and *Csf1r* mutants indicate that apoptosis is a far more important determinant of RGC number than microglia-mediated death (Fig 2B and 2D; S8C Fig). For astrocytes, by contrast, the opposite is true. Retinal astrocytes provide the first example, to our knowledge, of a CNS cell type that uses death by phagocyte as its primary mechanism of naturally occurring developmental death.

Microglial killing of astrocytes is highly regulated—it occurs within a specific developmental epoch and produces a relatively consistent population size by adulthood. What are the mechanisms that determine the timing and extent of astrocyte death? Our data suggest that microglia are driven to remove astrocytes due to signals derived from astrocytes themselves. A key indication that astrocytes produce pro-phagocytic signals is that microglia located in their vicinity (i.e., the RNFL) have a distinctive phagocytic phenotype. Moreover, several features of this phagocytic phenotype are expressed in a graded fashion that strongly correlates with the number of astrocytes present at any given age (see especially Fig 3E but also Figs 3C, 3F, 3G, 4C and 4D). These findings are consistent with a model whereby microglia sense astrocyte number to modulate their phagocytic capacity (Fig 8). In this model, the opening of the death period is driven by accumulation of excess astrocytes as they migrate into the retina, while its closing occurs when astrocyte numbers have dropped low enough to remove the phagocytic stimulus (Fig 8C). Other factors, of course, could also contribute to the timing and extent of astrocyte death. For example, intrinsic developmental changes could influence the timing of when microglia are competent to remove astrocytes. The model we provide here (Fig 8) should serve as a useful framework for exploring such possibilities and thereby dissecting the cellular and molecular mechanisms that regulate astrocyte population size.

### Compensatory astrocyte-mediated astrocyte death in the absence of microglia

When professional phagocytes are unavailable, non-professional phagocytes carry out engulfment functions such as clearing apoptotic corpses [61,62]. Here, we show that retinal astrocytes can substitute for microglia not only in this capacity but also in the engulfment of their astrocyte neighbors (Fig 7F and 7G). This activity is likely responsible for resumed astrocyte death following microglia ablation, as we did not find evidence for other potential death routes. Müller glial cells (or their radial glial progenitors) may also serve as non-professional phagocytes [65] (S7D Fig), but their contribution to astrocyte engulfment appears minor because approximately 80% of astrocyte debris was localized within other astrocytes.

What causes astrocytes to begin engulfing each other when microglia are absent? In the case of apoptotic corpse clearance, a key feature of the compensatory mechanism is that both microglia and non-professional phagocytes have intrinsic capacity to recognize and clear dying cells. However, non-professional phagocytes are far slower to begin engulfing an apoptotic cell after first encountering it [61]. This delay reserves non-professional phagocytosis for instances in which microglia cannot keep up. Once engulfment has begun, however, both cell types clear dead cells at similar rates [61]. For retinal astrocytes, the time course of compensatory death (Fig 6E; S8A and S8B Fig) suggests that precisely this mechanism could be at play. We show that the rightward shifts of these curves are due almost entirely to a delay in onset of cell loss; once loss begins, the dynamics of the decline phase are quite similar between wild-type and ablated retinas (S8B Fig). These data are well explained by a model in which astrocytes are slow to begin engulfing each other when the need first arises, but once removal is underway, it proceeds with a mostly normal time course. In this model, astrocytes, like microglia, are sensitive to pro-phagocytic astrocyte-derived cues (Fig 8), but they normally do not have time to act on these cues because microglia do so first. When microglia are absent, the latent propensity for developing astrocytes to engulf each other is unveiled.

### Relevance to astrocyte death in other CNS regions

Few previous studies have addressed quantitatively the extent of astrocyte death in the developing CNS. It was not known if astrocytes, like many types of neurons, are overproduced and then culled. Here, we show that retinal astrocyte populations, including RNFL astrocytes and Müller glia, follow such a pattern. Our data further suggest that brain astrocytes, in cortex and corpus callosum, behave similarly (S2 Fig).

Extrapolating from apoptosis assays, it was reported that astrocytes in cortex [23,25,26] and retina [27–29] were subject to developmental death. However, quantitative information about the rates of astrocyte apoptosis or its impact on total cell numbers was not included in these studies, leaving open the possibility that apoptosis has only a minor effect on population size. Indeed, in several of these studies, astrocyte apoptosis was remarkably infrequent [23,26]. A study in rat cerebellum showed that a large fraction—as many as 1%—of GFAP^+^ white matter cells are apoptotic at P7 [24]. At the time of this study, it was thought that these GFAP^+^ cells were astrocytes, but it is now known that they are actually multipotent neural progenitors rather than lineage-committed astrocytes [66]. Thus, astrocyte apoptosis rates in the cerebellum are likely much lower than originally proposed. Our retinal data indicate that microglia-mediated death is a far more significant contributor to astrocyte loss than apoptosis. It will be interesting to learn whether microglia also kill astrocytes in other regions of the CNS. As in the RNFL (Fig 3F and 3G), a transient population of Osteopontin^+^ phagocytic microglia is found in developing brain [46,67], raising the possibility that astrocytes could be targets of such cells. In this regard, it is noteworthy that microglia have recently been shown to kill astrocyte precursors in the amygdala to produce sex differences in cell number and behavior [68]. Unlike the retina (Fig 5), this microglial activity is dependent on complement and can be blocked by minocycline, suggesting that the molecular mechanisms may differ. It remains to be determined whether the brain and retina findings represent distinct phenomena or rather different implementations of a general microglia-mediated astrocyte death mechanism.

### Potential functions of astrocyte death in retinal development

Retinal astrocytes play a pivotal role in retinal angiogenesis, guiding vascular colonization of the RNFL during the perinatal period (P0 until approximately P8 in mice; Fig 1A; [22]). This guidance is accomplished both through direct physical patterning of growing vessels, as well as expression of key pro-angiogenic signals such as vascular endothelial growth factor (VEGF) [22,30,69]. Changes in the size of the astrocyte population would be expected to disturb angiogenesis by altering VEGF levels and by changing the pattern of the astrocyte network in a manner that could be propagated to vessels. Accordingly, when astrocyte numbers are artificially elevated through forced proliferation, patterning of both the astrocyte and vascular networks is perturbed. Crucially, these perturbations cause retinal bleeding during the early postnatal period [32]. This result highlights the importance of developmental mechanisms that maintain appropriate astrocyte numbers and the deleterious consequences when such mechanisms are dysregulated.

Here, we show that astrocytes are subject to developmental cell death during the period of RNFL angiogenesis. We propose that death may be part of a homeostatic mechanism that balances migration and proliferation to set the astrocyte population size. In this case, both increased proliferation [32] and decreased death (this study) would be expected to have similar effects. In *Csf1r* mutants, the magnitude of effects on the astrocyte network were smaller than in the proliferation study [32], likely due to the fact that we only partially blocked death. However, in both cases the number of astrocytes and the density of their arbor network were increased. Furthermore, in both cases retinal bleeding was observed during early postnatal development (Fig 6H and 6I; [32]). The many phenotypic similarities between these two mouse models suggest that bleeding in *Csf1r* mutants might be astrocytic in origin. In our study, we cannot exclude that bleeding was caused by an astrocyte-independent function of microglia, but in the study by Fruttiger and colleagues (1996), the manipulation was likely astrocyte specific. The presence of excess VEGF-producing astrocytes is a plausible cause of hemorrhage, because VEGF tends to promote endothelial cell proliferation and sprouting at the expense of quiescence, maturation, and barrier formation [70]. In order to stringently test the role of death in vascular development, it will be necessary to identify molecular manipulations that prevent microglia from killing astrocytes without eliminating microglia entirely. Despite extensive efforts (Fig 5), we were unable to identify such a manipulation in this study. However, we expect that this will ultimately become possible, thereby enabling definitive studies on the functional role of developmental astrocyte death.

## Methods

### Ethics statement

The Institutional Animal Care and Use Committees at Duke University (protocol numbers A005-16-01, A274-18-12) and the University of California at Santa Barbara (protocol number 443.2) reviewed and approved all experimental procedures involving animals at respective universities. Anesthesia was by isoflurane. Euthanasia was by overdose of isoflurane followed by decapitation.

### Procedures

#### Animals

Mice were maintained on a 12-hour light/dark cycle; food and water were freely and continuously available. All mouse strains used (Table 1) were maintained by continual backcrossing to C57Bl6/J, unless noted below. Upon receipt into our colony, each strain was genotyped for *Rd1* and *Rd8* retinal degeneration mutations; when necessary, these mutant alleles were bred out of the strain. As such, all animals used for experiments were free of these retinal degeneration mutations. For Cre-dependent reporter expression, we used both the *Rosa26*^*ai14*^ allele [71], which drives tdTomato expression, and the *Rosa26*^*iDTR*^ allele, which drives cell-surface expression of the human DTR; in the latter case, anatomy was assessed by antibody staining to DTR (see method details below). *Cx3cr1*^*CreER-ires-YFP*^ mice ([44]; also denoted *Cx3cr1*^*CreER*^) served three different purposes in this study: (1) microglia-specific Cre driver; (2) constitutive microglia-specific yellow fluorescent protein (YFP) expression; and (3) *Cx3cr1* loss-of-function allele (Fig 5).

**Table 1:** Key resources used in this study.

Reagent or Resource	Source	Catalog number
Antibodies		
Neurofilament: mouse, 1:500	BioLegend	SMI-32P
Pax2: rabbit, 1:200	Covance	PRB-276P
ChAT: goat, 1:200	Millipore	AB144P
Sox9: goat, 1:2,000	R&D Systems	AF3075
Sox9: rabbit, 1:4,000	Millipore	AB5535
Chx10: sheep, 1:300	Exalpha	X1180P
AP2α: mouse, 1:200	Develomental Studies Hybridoma Bank (Iowa, USA)	3B5
HB-EGF (i.e., DTR): goat, 1:500	R&D Systems	AF-259-NA
CC3: rabbit, 1:2,000	Cell Signaling	9661
GFAP: goat, 1:1,000	Abcam	AB53554
GFAP: mouse, 1:1,000	Sigma-Aldrich	G3893
PDGFRα: rat, 1:500	BD Pharmingen	558774
RBPMS: rabbit, 1:2,000	Gift of N. Brecha (UCLA) [72]	n/a
RBPMS: guinea pig, 1:2,000	Gift of N. Brecha lab (UCLA) [72]	n/a
GFP: chicken, 1:1,000	Life Technologies	A10262
Iba-1: rabbit, 1:1,000	Wako Chemicals	019–19741
Iba-1: goat, 1:750	Novus Biologicals	NB100
P2Y12: rabbit, 1:1,000	Anaspec	AS-55043A
CD68: rat, 1:500	BioLegend	137002
mCherry: rabbit, 1:3,000	Kerafast	EMU106
RFP: rat, 1:500	ChromoTek	5f8-20
CD45: rat, 1:500	BioLegend	103104
Ki67: rat, 1:3,000	Ebioscience	14-5698-80
Alexa Fluor 488 AffiniPure Donkey Anti-chicken: 1:1,000	Jackson ImmunoResearch	703-545-155
Alexa Fluor 488 AffiniPure Donkey Anti-rabbit: 1:1,000	Jackson ImmunoResearch	711-545-152
Alexa Fluor 488 AffiniPure Donkey Anti–guinea pig: 1:1,000	Jackson ImmunoResearch	706-545-148
Alexa Fluor 488 AffiniPure Donkey Anti-goat: 1:1,000	Jackson ImmunoResearch	705-545-147
Alexa Fluor 488 AffiniPure Donkey Anti-mouse: 1:1,000	Jackson ImmunoResearch	706-605-148
Alexa Fluor 647 AffiniPure Donkey Anti-rabbit: 1:1,000	Jackson ImmunoResearch	711-605-152
Alexa Fluor 647 AffiniPure Donkey Anti-goat: 1:1,000	Jackson ImmunoResearch	705-605-147
Alexa Fluor 647 AffiniPure Donkey Anti-mouse: 1:1,000	Jackson ImmunoResearch	715-605-151
Alexa Fluor 647 AffiniPure Donkey Anti-rat: 1:1,000	Jackson ImmunoResearch	712-605-150
Alexa Fluor 647 AffiniPure Donkey Anti–guinea pig: 1:1,000	Jackson ImmunoResearch	706-605-148
Cy3-AffiniPure Donkey Anti-rabbit: 1:1,000	Jackson ImmunoResearch	715-165-151
Cy3-AffiniPure Donkey Anti-Guinea Pig: 1:1,000	Jackson ImmunoResearch	706-165-148
Cy3-AffiniPure Donkey Anti-Goat: 1:1,000	Jackson ImmunoResearch	705-165-147
Cy3-AffiniPure Donkey Anti-Mouse: 1:1,000	Jackson ImmunoResearch	715-165-151
Cy3-AffiniPure Donkey Anti-Rat: 1:1,000	Jackson ImmunoResearch	712-165-150
Experimental Models: Organisms/Strains		
Mouse: Sun1-GFP (Gt(ROSA)26Sor^tm5(CAG-Sun1/sfGFP)Nat/J^)	Jackson Labs	021039
Mouse: Aldh1l1-CreER (Tg(Aldh1l1-cre/ERT2)1Khakh/J)	Jackson Labs	029655
Mouse: Aldh1l1-EGFP (Tg(Aldh1l1-EGFP)OFC789Gsat)	MMRRC	011015
Mouse: CD-1 (Crl:CD1(ICR))	Charles River	022
Mouse: SJL/J	Jackson Labs	000686
Mouse: C57Bl6/J	Jackson Labs	000664
Mouse: *Cx3cr1*^*CreER-ires-YFP*^ (Cx3cr1tm2.1(cre/ERT2)Litt)	Jackson Labs	021160 [44]
Mouse: *GFAP-Cre* (Tg(GFAP-cre)25Mes)	Jackson Labs	004600 [73]
Mouse: *Rosa26*^*Ai14*^ (*Gt(ROSA)26Sor*^*tm14(CAG-tdTomato)Hze/J*^)	Jackson Labs	007914 [71]
Mouse: *Rosa26*^*iDTR*^ (*Gt(ROSA)26Sor*^*tm1(HBEGF)Awai*^)	Jackson Labs	008040 [74]
Mouse: *Bax*^*flox*^ (*Bax*^*tm2Sjk*^)	Jackson Labs	006329
Mouse: *Brn3b*^*Cre*^ (*Pou4f2*^*tm1(cre)Bnt*^)	Jackson Labs	030357
Mouse: *Itgam*^*–/–*^(*Itgam*^*tm1Myd*^)	Jackson Labs	003991 [75]
Mouse: *Mertk*^*–/–*^(*Mertk*^*tm1Grl*^)	Jackson Labs	011122 [76]
Mouse: *Syk*^*flox*^(*Syk*^*tm1*.*2Tara*^)	Jackson Labs	017309
Mouse: *Cx3cr1-Cre (Tg(Cx3cr1-cre)MW126GSat)*	S. D. Bilbo (Duke) [57]	
Mouse: *Myd88*^*flox*^(*Myd88*^*tm1Defr*^)	Jackson Labs	008888
Mouse: *Csf1r*^*flox*^(*Csf1r*^*tm1*.*2Jwp*^)	Jackson Labs	021212 [77]
Mouse: *Csf1r*^*null*^	generated from *Csf1r*^*flox*^ allele (see Methods)	n/a
Mouse: *Actin-Cre* (*Tmem163*^*Tg(ACTB-cre)2Mrt*^/J)	Jackson Labs	003376
Software and Algorithms		
GraphPad Prism 7.0	GraphPad	RRID:SCR_002798
ImageJ 1.50e	ImageJ	PMID 22930834
WinDRP v1.6.4	T. Euler (Tübingen)	n/a
Olympus cellSens	Olympus Lifescience	n/a
NIS-Elements	Nikon	n/a
Other		
Tamoxifen	Sigma-Aldrich	T5648
Corn Oil	Welch, Holme & Clark Co.	8001-30-7
Diphtheria toxin	Sigma-Aldrich	D0564
16% Paraformaldehyde	Electron Microscopy Sciences	15710
Glutaraldehyde solution	Sigma-Aldrich	G5882
Normal Donkey Serum	Jackson ImmunoResearch	017-000-121
Cellulose Membrane Filter	Millipore	HABG01300
Fluoromount G	SouthernBiotech	0100–01
Nail Polish	Electron Microscopy Sciences	72180
Minocycline Hydrochloride	Sigma-Aldrich	M9511
Hoechst 33258	Invitrogen	H21491
Isothesia: Isoflurane	Henry Schein	11695–6776
Tissue Freezing Medium	VWR	15148–031
LE Quick Dissolve Agarose	GeneMate	E-3120-500

Abbreviations: CC3, cleaved-caspase 3; ChAT, choline acetyltransferase; DTR, diphtheria toxin receptor; GFAP, glial fibrillary acidic protein; GFP, green fluorescent protein; HB-EGF, heparin binding epidermal growth factor-like factor; MMRRC, mutant mouse regional resource center; PDGFR, platelet-derived growth factor receptor; RFP, red fluorescent protein; YFP, yellow fluorescent protein

To generate *Csf1r* null mutants (*Csf1r*^*–/–*^), *Actin-cre* animals were crossed to *Csf1r*^*flox*^ animals to allow for germline recombination of *Csf1r*. We observed that homozygous *Csf1r* null mutations are lethal at birth on a pure C57Bl6/J background; therefore, we used a hybrid C57Bl6-SJL strategy to generate viable *Csf1r* mutants. In this strategy, two sets of breeders were used for experimental matings. One set (denoted B6-*Csf1r*^*+/–*^) was produced by continual backcrossing of the *Csf1r* null allele to C57Bl6/J; the other set was produced by outcrossing the B6-*Csf1r*^*+/–*^mice to SJL/J, thereby generating F1 hybrids of B6 and SJL that carried the *Csf1r* null allele (denoted B6SJL-*Csf1r*^*+/–*^). Experimental litters were generated by crossing B6-*Csf1r*^*+/–*^to B6SJL-*Csf1r*^*+/–*^. Mutant progeny routinely survived to approximately P21 without special husbandry. Note that, because of this special breeding strategy, it is exceedingly difficult to deploy Cre-lox reporters in the *Csf1r* background: because Cre and reporter lines are not available on an SJL background, crossing them into the *Csf1r* background would likely result in neonatal lethality. For all experiments, controls were *Csf1r*^+/+^ littermates, and at least one eye per animal was stained for microglia to confirm their absence.

To study the role of TAM receptors in astrocyte death, we studied *Mertk*^–/–^mutants. Microglia also express a second TAM receptor, *Axl*, and in brain, both genes participate in phagocytosis. However, loss of *Mertk* alone is sufficient to impair removal of dead or virus-infected cells [16,51]. Therefore, *Mertk*^–/–^single mutants should show at least a partial phenotype if TAM receptors have a function in retinal astrocyte death.

#### Tamoxifen administration

To induce CreER‐mediated recombination, 100 μg of tamoxifen, an estrogen receptor ligand, was administered via intraperitoneal (IP) injection to neonatal mice. Tamoxifen powder was dissolved in corn oil at a concentration of 20 mg/mL by sonicating in a room temperature water bath for 30 minutes. For *Cx3cr1*^*CreER*^ animals, injections were administered at P2, P5, and P7 for DTR experiments and at P0 or P1 for *Syk*^*flox/flox*^ experiments. For *Aldh1l1-CreER* animals, injections were administered at P1. Both control and experimental animals received tamoxifen injections in all experiments that required CreER-mediated recombination, unless otherwise noted.

#### Diphtheria toxin–mediated microglia ablation

For microglia ablation experiments, the *Rosa26*^*iDTR*^ line [74] was crossed to two different microglial Cre drivers: (1) *Cx3cr1*^*CreER-ires-YFP*^ (see above) and (2) a *Cx3cr1-Cre* line generated using a bacterial artificial chromosome transgenesis strategy [57]. Most experiments were performed on the *Cx3cr1*^*CreER*^ background. To establish an ablation paradigm for neonatal mice, we first tested the specificity, efficacy, and temporal characteristics of the *Cx3cr1*^*CreER*^; *Rosa26*^*iDTR*^ ablation paradigm in neonatal retina. Administration of tamoxifen at P2 induced cell type–specific expression of DTR in nearly all microglia by P4 (S5A Fig; 97.41 ± 0.52% of microglia were DTR^+^; 100% of DTR^+^ cells were microglia; *n* = 580 cells from 2 mice, mean ± SEM). DTR-expressing mice, but not littermate controls lacking either the CreER or the DTR transgenes, were susceptible to microglia ablation upon administration of diphtheria toxin. A single 80-ng dose depleted microglia to approximately 10% of control levels within 2 days (Fig 6B; S5B Fig). Depletion was confirmed by staining for 3 different microglial markers: Iba-1, CD45, and YFP driven by the *Cx3cr1*^*CreER*^ allele (Fig 7E, S8E and S6A Figs). Microglia were specifically affected—we did not observe any effects of diphtheria toxin on overall retinal histology (S5D Fig; [30]). By 4 days postinjection, however, microglia had partially repopulated the retina, returning to approximately 50% of control levels, only approximately 60% of which expressed DTR (S5B and S5C Fig).

To ablate microglia for longer periods of time, we developed a regime for repeated tamoxifen and diphtheria toxin administration; this successfully prevented return of microglia for the entire administration period—as long as P6–P14 (Fig 6A and 6B). In this regime, tamoxifen was administered as described above to induce DTR expression; diphtheria toxin was then administered at P4, P6, P8, P10, and P12 to allow for continuous depletion of microglia. Diphtheria toxin, dissolved in 1X PBS was administered IP at 80 ng per dose; we previously found this dose to be optimal for ablation without off-target effects in neonatal pups [30]. For most experiments, the control group was littermates that were injected with both tamoxifen and diphtheria toxin but did not inherit one of the two key transgenes (i.e., *Cx3cr1*^*CreER*^ or *Rosa26*^*iDTR*^). In initial experiments, we compared control animals of this type with those that received only tamoxifen but no diphtheria toxin (or, in two cases, animals that received neither; these animals are denoted by gray dots with black outlines in S6B Fig). There was no difference between these control groups in total astrocyte number counts at P8 and P10 (S6B Fig), so data from both control groups were ultimately combined for final analyses (Fig 6; S6 Fig). For DTR-mediated microglia ablation experiments in the *Cx3cr1-Cre* background, the same diphtheria toxin dosage described above was administered to both control and experimental animals at P6 and P8. Control animals were littermates that lacked either the *Cx3cr1-Cre* or *Rosa26*^*iDTR*^ transgenes.

#### Minocycline administration

For minocycline experiments, CD-1 mice were IP injected once daily with minocycline (Sigma; 50 mg/kg) or vehicle (10 mM Tris-HCL) from P4 to P9. Dosage was determined based on previously published studies [78].

#### Histology

For retina histology, mice were typically anesthetized and euthanized by decapitation, followed by immediate enucleation. Whole eyes were fixed on ice in 4% paraformaldehyde (PFA) in 1X PBS for between 1.5 and 2 hours, and subsequently washed twice (5 minutes each) in 1X PBS. In some cases, mice were perfused transcardially with 4% PFA/1X PBS prior to eyeball postfixation, as above. Eyes were either processed for histology immediately following fixation or stored at 4°C in 0.02% sodium azide in 1X PBS until the time of tissue processing. Retinas were prepared for cryosections or whole-mounts as previously described [79]. Briefly, for retinal sections, the lens was extracted from the eyecup and vitreous removed. Eyecups were then cryoprotected in 30% sucrose for at least 2 hours prior to embedding in Tissue Freezing Medium. After freezing, 20 μm cryosections were collected with a Microm HM 550 cryostat. For whole-eye sections, vitreous structures were maintained by leaving both retina and lens within the eyecup and puncturing the cornea with a 30-gauge needle followed by a slight elongation of the puncture with iris scissors to allow for thorough cryoprotection. After cryoprotection, whole eyes were embedded in Tissue Freezing Medium, and 20 μm cryosections were obtained as previously described. Whole-mount retinas were obtained by also extracting lens and vitreous, in addition to detaching the retina from the eyecup.

For brain histology, mice were transcardially perfused with 1X PBS followed by 4% PFA in 1X PBS. After perfusion, mice were decapitated and whole brains were removed and postfixed overnight at 4°C in 4% PFA in 1X PBS and then washed three times (30 minutes each) in 1X PBS. For sectioning, whole brains were embedded in 2% agarose gel, and 80 μm horizontal sections were generated on a Leica VT 1200 S vibratome. Sections containing both cortex and corpus callosum were subsequently used for quantification of astrocyte density.

#### Immunohistochemistry

Retinal tissue was blocked in a solution of 3% normal donkey serum, 0.3% Triton-X, and 0.02% sodium azide in 1X PBS at room temperature (30 minutes for retinal sections; 2 hours for whole-mount retina). After blocking, tissue was incubated with primary antibodies in the aforementioned blocking solution either overnight (retinal sections) or for 5 days (whole-mount retina). Tissue was washed after primary antibody incubation at least three times in 1X PBS. Secondary antibodies and Hoechst 33258, in a solution of 0.3% Triton-X in 1X PBS, were then applied for either 2 hours at room temperature (retinal sections) or overnight at 4°C (whole-mount retinas). Finally, tissue was washed at least three times at room temperature before preparing samples for image acquisition. Primary and secondary antibodies used in this study are listed in Table 1.

#### Image acquisition and processing

Following immunohistochemistry, whole retinas were placed in a dish of 1X PBS and four radial cuts separated by 90 degrees were made, with each cut extending approximately one third of the way from the edge of the retina to the optic nerve head. Cut retinas were placed ganglion cell–side up on nitrocellulose filter paper and carefully laid flat with a paintbrush before mounting on a slide. Prior to imaging, slides were coverslipped with a layer of Fluoromount G mounting media between the sample and coverslip. The coverslip was held in place with a layer of clear nail polish applied at the seam between the edge of the coverslip and the slide. A Nikon A1 or an Olympus FV300 confocal microscope was used to image sections and whole-mounts. To produce large field-of-view images, tiled frames were acquired with a 20× air objective via the Nikon A1 confocal or an Olympus IX81 epifluorescence microscope, using automated software supplied by the microscope manufacturer. Images were then stitched into a single image using Olympus or Nikon software. For section and whole-mount images, Z-stacks were collected at a Z-resolution of 0.3–1 μm; 1.5 μm Z-resolution was used for tilescan Z-stacks. Image stacks were imported to Fiji [80] for processing and analysis. Images selected for display were first maximum projected to a single plane prior to de-noising by median-filtering (2.0 pixel radius for most images; in rare instances, a pixel radius >2.0 and up to 10.0 was utilized). The portion of the stack selected for maximum-intensity projection was determined by the Z-volume of the structure to be depicted in the final image. Assembly of color channels as well as minor adjustments to brightness and contrast were also made in Fiji. For data analysis and quantification, only original stacks and not Z-projections were used, unless otherwise noted.

#### Plastic sections and electron microscopy

Immediately following enucleation, whole eyes were fixed in 2% PFA + 2% glutaraldehyde in 1X PBS for 1 hour at room temperature and then overnight at 4°C. Eyeballs were then washed twice in 1X PBS and immersed in 2% osmium tetroxide in 0.1% cacodylate buffer, dehydrated, and embedded in Epon 812 resin. For light microscopy, semi-thin sections of 0.5 μm were prepared and counterstained with 1% methylene blue. For electron microscopy, thin sections of 65–75-nm thickness were collected on a Leica EM CU7 and counterstained with a solution of 2% uranyl acetate and 3.5% lead citrate, and examined using a JEM-1400 transmission electron microscope at 60 kV. An Orius 1000 charge-coupled device camera was used to collect images. Astrocyte nuclei were identified by their localization proximal to RGC nerve fibers, the oblong shape and size of their nucleus, and their unique heterochromatin pattern. As pericytes also have a nuclear morphology similar to that of astrocytes, we distinguished between these cell types based on the characteristic tight association between pericytes and endothelial cells. RBCs were identified by their characteristic irregular shape and electron-dense staining profile.

### Quantification and statistical analysis

#### Statistics

Error bars are expressed as mean ± SEM, unless otherwise noted. For all analyses, alpha was set to 0.05. Statistical parameters (i.e., sample size, statistical and post hoc tests, and statistical significance) are reported in every figure or figure legend. All *t* tests were two-tailed. One-way, two-way, or three-way ANOVAs without matching, followed by the appropriate post hoc test, were utilized. All ANOVA results are given in S1 Table. Asterisks denote statistical significance (*p ≤ 0.05; **p ≤ 0.01; ***p ≤ 0.001; ****p ≤ 0.0001) in every figure. Data were analyzed using GraphPad Prism v7 software.

#### Quantification of astrocyte density and total astrocyte number

For retina, we sampled NFL astrocyte density from central, middle, and peripheral regions (Fig 1E). Typically, 3–4 60× images per retinal region were acquired for the analysis, although in rare cases only 2 images were available. Only retinal locations free of damage were sampled. These locations were chosen using anatomical features of the retina so as to maintain consistency in sample selection. Central images were chosen by sampling retinal tissue just proximal to where blood vessels first bifurcate upon exiting the optic nerve head into the retina. Middle images were acquired at the approximate midpoint of the line connecting the edge of the retina to the optic nerve head. Finally, peripheral images were acquired approximately 2 to 3 astrocyte cell bodies away from the edge of the retina. Astrocyte density for each image was quantified by hand using ImageJ/FIJI software to mark each cell. The average density per region was then converted into an overall weighted average density based on the total retinal area within each sampled region (weighting: central = 11%, middle = 33%, peripheral = 56%; see Fig 1E and [30]). This weighted average was multiplied by retina area to obtain a value for the estimated total astrocyte number. Retina areas were measured in FIJI by drawing perimeters with the Freehand selection tool on tilescanned images of whole-mount retinas.

Total Müller glia counts were obtained from retinas of C57Bl6/J mice. Images were taken from retinal whole-mounts at 60× magnification. As the density of Müller cells does not vary by eccentricity [81], the complex sampling procedure used for astrocytes was not necessary. All images used in the analysis were derived from an eccentricity midway between the optic nerve and the peripheral edge of the retina. Cells were counted by hand with ImageJ/FIJI software. Averages were subsequently multiplied by retinal area to obtain a value for estimated total Müller glia number.

For brain, Aldh1l1-GFP mice were used to quantify both cortical and callosal astrocytes from horizontal brain sections (80 μm in thickness). Counts were made using unprojected Z-stacks through the region to be analyzed. For corpus callosum, only astrocytes in the decussation of the callosum were included. Astrocyte density measurements for a given image were obtained by manually measuring the area of the corpus callosum and normalizing astrocyte counts to this area. For cortex quantification, images were acquired in motor cortex from sections containing corpus callosum. All astrocytes within a given 3D volume were counted.

#### Modeling

Overall strategy: To estimate the contribution of apoptosis and phagocytosis to developmental astrocyte loss, a model originally formulated to estimate developmental cortical neuron death was applied to our system [37]. This model predicts the number of cells that remain at the end of the death process (*N*_*T*_) based on the rate at which cells are dying and the starting number of cells (*S*). The rate of death is determined by two parameters: (1) the fraction of the cell population that is visibly dead or dying on a given day (*D*_*n*_) and (2) the amount of time for which a dead cell is visible on day *n* (*V*_*n*_; this can also be thought of as a clearance time). By assuming that *D*_*n*_ and *V*_*n*_ are constant (i.e., reflecting an average across all days of development), we were able to simplify the model to the following equation:

$$N_{T}={S(1-D(\frac{24}{V}))}^{n}$$

If the starting number and final number of cells, as well as the fraction of dead cells in a given death process, are known, clearance time can be determined by solving for *V*:

V=24D1−(NTS)1n

To validate that this model can accurately estimate cell death under the conditions of our assumptions, we utilized it to predict *V* (visibility, or clearance time) for RGCs, as this is a cell population known to undergo apoptosis during normal development [38]. We used a previously published rat RGC developmental data [35] to carry out this validation. Note that in the original publication, data for RGC counts and total number of degenerating neurons in the ganglion cell layer (GCL) were listed with ages 0 to 10 days after birth. These ages are related to the date of injection for RGC retrograde labeling with horseradish peroxidase (HRP), while actual RGC quantification was done 18 hours postinjection—therefore, day 0 is actually P1, day 3 is actually P4, and so on. Thus, for our purposes, we are calling “0 to 10 days after birth” now “P1 to P11.” Based on this paper [35], the following parameter values were used for the model (also see S3D Fig): *D* was the average percentage of neurons in the GCL found to be pyknotic between P1 and P11 (*D* = 0.005375, or 0.5375%); *S* = 200,000 RGCs (P1); *N*_*T*_ = 117,000 RGCs (P11); and *n* = 10 days (P1 to P11). While *D* in this case is not specific to RGCs (given that amacrine cells also reside in the GCL), we made the assumption that all neurons of the GCL undergo a similar rate of degeneration and thus treat *D* as if it were the rate of RGC death. Based on the fact that our predicted *V* for RGCs (Fig 2B) was in good alignment with clearance rate measurements made independently by others [38–41], we concluded that the assumptions underlying our model were sound. As such, we proceeded to use the model to investigate astrocyte death.

Modeling apoptosis as an astrocyte death mechanism: To estimate the contributions of apoptosis to astrocyte death, we denoted *S* to be the average number of astrocytes found at P5 (thus, *S* = 26,987 cells). This age was chosen for several reasons. First, P5 is when astrocyte numbers are at their highest. Additionally, astrocytes have finished migratory colonization of the retina by this age, eliminating the need to account for cellular migration when predicting the relationship between total astrocyte numbers and the contributions of cell death. *D* was either the experimentally determined overall average (*D*_*avg*_ *=* 0.000583, or 0.0583%) percentage of CC3^+^ astrocytes during the P5 to P14 period, or the maximum CC3^+^ percentage observed for any given age (*D*_*high*_ = 0.001622, or 0.1622%) (S3C Fig). *n* was set to 9 (i.e., the length of the P5–P14 death period). These three parameters were held constant for all three analyses described below.

We used the model to address three different questions related to astrocyte apoptosis. First, we asked what value of clearance time *V* would be required to account for the change in astrocyte number between P5 (*S*) and P14 (*N*_*T*_ = 6,513 cells) if the death rate were the observed *D*. The computed value of *V* (Fig 2C, blue curve) was on the order of approximately 5 minutes. In a second permutation of the model, we asked whether the observed death rate *D* could predict the observed *N*_*T*_ if the clearance time *V* were set to a biologically plausible value. For this purpose, we used the clearance time for apoptotic RGCs (2.47 hours) as *V*. The outcome of this calculation is the red curve plotted in Fig 2C.

Third, we used the model to estimate the total number of astrocytes that died by apoptosis within a single day in wild-type retina. This number was then used to estimate the expected CC3^+^ astrocyte density at P6 in a situation where apoptotic corpses could not be cleared (e.g., in mice lacking microglia). To make the total number estimate, the same model parameters were used as in the second scenario above to generate an astrocyte numbers time course (e.g., Fig 2C, red line). The number of astrocytes lost each day was determined from the difference between astrocyte numbers generated by the model for day *N* and day *N* + 1 and repeated for all sequential pairs of days. We then calculated the average number of astrocytes lost per day and multiplied this average by 6 days to arrive at an estimate of astrocytes lost to apoptosis between birth and P6. To put confidence bounds on this estimate, we determined the 95% confidence interval of our CC3^+^ astrocyte number measurement (S3C Fig) and performed the same analysis again using the upper, mean, and lower confidence interval values for the death rate *D* parameter. Thus, the calculation of “astrocytes lost” between P0 and P6 was ultimately performed three times with three different *D* values (*D*_*mean*_ = 0.03%; *D*_*min*_ = 0.02%; *D*_*max*_ = 0.06%), generating a “mean” as well as an upper and lower limit to our estimate of astrocytes lost. Finally, we used these values to calculate the expected density of CC3^+^ astrocytes at P6 if none of the apoptotic astrocytes that died prior to P6 were ever cleared (Fig 7B, blue line). The conversion from total number to density was made by dividing by the average retinal area at P6.

Modeling phagocytosis as an astrocyte death mechanism: To calculate *V* as it relates to the contributions of phagocytosis to astrocyte death, we again denoted *S* to be the average number of astrocytes found at P5 (thus, *S* = 26,987 cells). *D* was the experimentally determined average percentage of astrocytes found to be fully enveloped by microglial processes during the P5–P14 period (thus, *D* = 0.00556, or 0.556%). As before, *N*_*T*_ was the average number of astrocytes found at P14 (thus, *N*_*T*_ = 6,513 cells) and *n* was 9 (P5 to P14).

Modeling astrocyte cannibalism as an astrocyte death mechanism in the absence of microglia: To calculate *V* as it relates to the contributions of astrocyte cannibalism to astrocyte death in the absence of microglia, we denoted *S* to be the average number of astrocytes present at P6 in *Csf1r* null retina (thus, *S* = 30,063 cells). *D* was the experimentally determined average percentage of astrocytes found to contain Pax2^+^ puncta at P6 in *Csf1r* null retinas (thus, *D* = 0.0168, or 1.68%) and was held constant for all ages. Because astrocytes were identified using PDGFRα in the Pax2^+^ puncta analysis, it was not possible to truly know whether multiple debris were contained within one astrocyte or if each debris was confined to unique astrocytes. Thus, it was assumed that there was a 1–1 ratio between the number of debris and the number of astrocytes containing debris. Finally, *N*_*T*_ was the average number of astrocytes found at P14 in *Csf1r* null retina (thus, *N*_*T*_ = 14,247 cells) and *n* was 8 (P6 to P14).

#### Microglia–astrocyte interaction analysis

Images were collected from retinal whole-mounts by sampling central, middle, and peripheral retina as described above. Microglial morphology was revealed in *CX3CR1*^*CreER-ires-YFP*^ mice using anti-GFP immunohistochemistry. Astrocytes were classified as being touched, partially enveloped, or fully enveloped by a microglial process, based on assessments of their somata labeled with either Pax2 or Sox9. The term “enveloped” refers only to the cell body. Any astrocyte soma found to be contacted by a microglial process was labeled as “touched.” If an astrocyte soma was found to have at least 50% of its circumference enveloped by a microglial process, it was labeled as “partially enveloped.” Astrocyte somata were counted as “fully enveloped” if the entire circumference of the astrocyte soma was surrounded by a microglial process.

#### Lysosome index

To quantify microglial lysosomal content, 60× confocal Z-stacks, which captured the entire depth of the RNFL plus GCL (approximately 12 μm total, 1 μm Z-steps) were obtained from retinal whole-mounts stained for Iba-1 (to identify microglia) and CD68 (to identify their lysosomes). Sampling methodology from central, middle, and peripheral retina was as described above. Laser settings were individually adjusted for each image to ensure that under- and overexposure of CD68^+^ pixels did not occur. Stacks were processed into maximum intensity Z-projections using Fiji software. On rare occasions, there were images that contained CD68^+^ endothelial cells—such images were discarded in this analysis. In Fiji, CD68^+^ pixels were segmented using thresholding (settings: Li, black and white, dark background). The ImageJ function “Analyze Particles” was used to determine the percentage of image area occupied by CD68^+^ pixels (Analyze Particles settings: size 0–Infinity, analyzed in pixel units, circularity 0.00–1.00, include holes). The number of microglia within the given image was quantified and the number of CD68^+^ pixels normalized to the number of microglia to obtain a Lysosome Index for the given image. For cross-developmental comparison between Lysosome Index and total astrocyte number, both measures were normalized to maximum values and plotted on a 0–100 scale using GraphPad Prism software.

#### Debris quantification

Pax2^+^ or tdTomato^+^ astrocyte debris were classified as engulfed by microglia only if the debris were found to be enclosed on all sides by microglia or their phagosomes. This was determined by examining confocal Z-stacks encompassing the entire microglial cell (Z-step 0.4–1.0 μm). A similar analysis was performed to assess astrocytic engulfment of Pax2^+^ debris in *Csf1r* mutants. For wild-type Pax2^+^ debris studies, microglia were labeled by staining for anti-GFP in *Cx3cr1*^*CreER-ires-YFP*^ mice (P3, P5, P10, and P14). For *Csf1r* mutant studies, the strain was as described in the “Animals” section above (P6). Note that different lots of the Pax2 antibody supplied since 2015 have exhibited higher background, making them unsuitable for debris analysis. In the lots we received prior to that time, background was extremely low, making identification of debris quite simple. We conducted follow-up studies using tdTomato in order to rule out the possibility that the debris was an artifact of the original Pax2 antibody lots.

#### Astrocyte network analysis

Images were collected from GFAP-stained retinal whole-mounts by sampling central, middle, and peripheral retina as described above. For quantification of astrocyte network coverage, 60× confocal Z-stacks, which captured the entire depth of the RNFL plus GCL (approximately 12 μm total, 1 μm Z-steps), were processed into maximum intensity Z-projections using Fiji software. Only central and middle retina were sampled, as GFAP expression is low in peripheral retina at P10. Total network coverage was quantified by measuring the percentage of GFAP negative space within a given ROI and subtracting this percentage from 100 to obtain the percentage of space occupied by the GFAP astrocyte network. GFAP negative space was segmented using automatic thresholding (settings: Triangle), followed by application of the ImageJ function “Analyze Particles” (Analyze Particles settings: size 0–Infinity, analyzed in pixel units, circularity 0.00–1.00). Data were normalized to control by litter prior to statistical analysis due to litter-to-litter variability.

For quantification of astrocyte network regularity, 60× confocal Z-stacks through the RNFL and GCL were acquired from P10 retinas stained with antibodies to GFAP and Pax2 or Sox9. Using Fiji software, a dot was manually placed at the center of each cell to generate X–Y coordinates. These coordinates were used to produce Voronoi domain regularity indices using Fiji software, as previously described [82].

#### Software

Software utilized in the current study are listed in Table 1.

## Supporting information

S1 Fig Evaluation of possible mechanisms underlying developmental astrocyte loss. (A) Astrocytes do not migrate out of the RNFL during the death period. Whole-eye cross section from P10 mouse, stained for GFAP to label astrocytes and counterstained with Hoechst. Astrocytes are only found within the retina (in the RNFL; red arrow) and have not migrated into extraretinal spaces such as the vitreous or lens. P10 was chosen for this analysis because astrocyte numbers have declined substantially by this age, so if migration was a major cause of astrocyte loss, we should have seen many astrocytes in non-retinal regions by this time. (B) Astrocyte lineage tracing during the period of astrocyte loss, using *GFAP-cre* mice crossed to a Cre reporter (*Rosa26*^*iDTR*^). P9 retinal cross sections were immunostained with anti-DTR to reveal cells that experienced Cre activity, and for Sox2 as a marker of astrocytes and Müller glia. Cre reporter expression is only found within two astrocytic cell types: Sox2^**+**^ astrocytes of the RNFL (yellow arrows) and Sox2^**+**^ Müller glia within the INL (black arrow). This finding demonstrates that GFAP^**+**^ astrocytes do not transdifferentiate into a non-astrocytic cell type. Because astrocyte precursors activate Cre expression as early as P0–P1 [30], we would expect to see reporter-positive neurons by P9 if transdifferentiation were responsible for the decline in astrocyte numbers. If RNFL astrocytes were transdifferentiating into Müller glia, we would expect to see some reporter-positive cells migrating between the RNFL and the INL, where Müller cells reside (black arrow). However, no migrating reporter-positive cells were observed. (C) Astrocyte lineage tracing using *GFAP-cre* and a nuclear Cre reporter (Sun1-GFP). Representative en face images are shown. At P4, the vast majority (91.37% ± 3.12%) of Sox9^**+**^ astrocytes are also GFP^**+**^ (*n* = 2,819 astrocytes, *N* = 2 animals). This result demonstrates that Cre is active in the majority of astrocytes at early stages of their differentiation, providing an important control for the P9 lineage tracing shown in (B). (D) Anatomy of the retinal astrocyte network at P5, as shown in en face images stained for the Cre reporter tdTom driven by *GFAP-cre*. Scale bars, 50 μm (B, C, D); 100 μm (A). DTR, diphtheria toxin receptor; GFAP, glial fibrillary acidic protein; GFP, green fluorescent protein; INL, inner nuclear layer; RNFL, retinal nerve fiber layer; tdTom, tdTomato.
(TIF)

S2 Fig Developmental changes in the size of other astrocyte populations. (**A**) Representative images of Aldh1l1-GFP^+^ astrocytes in corpus callosum (top) and somatosensory cortex (bottom) viewed in horizontal sections. Yellow dashed lines denote boundaries of corpus callosum. (**B**) Quantification of astrocyte density across development for cortex and corpus callosum. Statistics: one-way ANOVA followed by Sidak multiple comparisons test. Also see S1 Table. *p = 0.0127 (P12 versus P26); ****p <0.0001 (P4 versus P26). (**C**) Representative images of Müller glia nuclei, shown in en face images from retinal whole-mounts stained for Sox9. (**D**) Quantification of total Müller glia numbers across development (see Methods). Statistics: two-tailed *t* test (*p* = 0.0149). Error bars, mean ± SEM. Sample sizes are denoted by data points on graphs. For data plotted in graphs, see S1 Data. Scale bars, 10 μm (C); 100 μm (A).
(TIF)

S3 Fig Assessment of astrocyte apoptosis. (A) Confocal images illustrating astrocyte and RGC densities in control and *Bax* mutant mice. Images similar to these were used for quantification shown in Fig 2D and 2E. Sox9^**+**^ astrocytes did not differ in density between wild-type controls and cell type–specific *Bax* mutants (left panels). More RBPMS^**+**^ RGCs are evident following *Bax* deletion in RGCs (*Brn3b-cre*) but not following *Bax* deletion in astrocytes (*GFAP-Cre*). (B) Representative electron micrographs showing ultrastructural morphology of retinal astrocytes at P5. Red arrows = astrocyte nuclei. Note the absence of anatomical features typical of various cell death pathways, such as (1) apoptosis (condensed nuclei), (2) necrosis (swollen cells/nuclei), and (3) autophagy (vacuolization) [34]. Analysis was performed on two sections from each of five animals; overall, 69 images were analyzed, each of which typically contained 1–2 astrocytes and occasionally contained >3 astrocytes. (C) Quantification of retinal astrocytes expressing CC3 across development. ***D***_***avg***_ = average death rate; ***D***_***high***_ = highest death rate (both values are utilized in the model found in Fig 2C; see **Methods**). Overall values for the columns “Astrocytes Counted” and “# Astrocytes CC3+” are totals; overall values for the columns “#CC3/100 Cells (Absolute)” and “#CC3/100 Cells (Average)” are averages. (D) Data from Perry and colleagues (1983) quantifying rat RGCs and the number of pyknotic GCL neurons across development. These data were utilized in the model found in Fig 2B. ***D***_***avg***_ = average death rate (see **Methods**). Scale bars, 50 μm (A, Astrocytes); 25 μm (A, RGCs); 2 μm (B). CC3, cleaved-caspase 3; GCL, ganglion cell layer; *GFAP*, glial fibrillary acidic protein; RGC, retinal ganglion cell.
(TIF)

S4 Fig Astrocyte debris is internalized by microglia. (**A**) An example of a Pax2^+^ debris punctum (arrow), similar to Fig 4A, within microglial cell labeled by *Cx3Cr1*^*CreER*^ YFP transgene. Three-dimensional reconstruction of the confocal stack was used to generate orthogonal views (XZ and ZY) through the debris particle. From all angles, it is evident that the debris is contained within the GFP^+^ microglial cell. (**B**) The same tdTomato^+^ astrocyte debris shown in Fig 4B, accompanied by 3D reconstruction of confocal Z-stack. Orthogonal views through indicated debris particle (arrow) reveal that microglial phagocytic cup surrounds the debris. Scale bars, 5 μm (A, B, orthogonal views); 10 μm (B, en face view). YFP, yellow fluorescent protein.
(TIF)

S5 Fig Ablation of microglia via inducible DTR system does not result in retinal abnormalities. (**A**) Representative image of microglia from P4 *Cx3cr1*^*CreER-ires-YFP*^*;Rosa26*^*iDTR*^ retina, stained for anti-GFP and anti-DTR. Mice received one dose of TMX at P2 to induce expression of DTR. Virtually all GFP^+^ microglia are also DTR^+^. See Results for cell count data. (**B**) Quantification of RNFL microglia density following a single round of TMX and DT, administered at the indicated time points (gray, red arrows). In *Cx3cr1*^*CreER*^*; Rosa26*^*iDTR*^ animals (red data points), microglia were largely eliminated by 2 days post-toxin, but significant repopulation was seen by 4–5 days post-toxin. Based on this finding, we administered diphtheria toxin at 2-day intervals in our long-term ablation paradigm (Fig 6B). Gray data points: control data from non-littermate animals from the *Cx3cr1*^*CreER-ires-YFP*^ background for comparison; these animals did not receive TMX or diphtheria toxin. (**C**) Quantification of DTR expression by spared microglia in the same ablated animals shown in (B). At 2 days post-toxin, few microglia remain (B), but a substantial fraction of these are DTR negative. The DTR-negative fraction is even higher by 6 days postinjection, suggesting that much of the repopulation is performed by microglia that escaped CreER-mediated DTR expression. This finding led us to conclude that long-term microglia ablation would require multiple TMX injections (as in the paradigm described in Fig 6B). (**D**) Representative retinal cross sections from P10 diphtheria toxin ablated mice or their littermate controls. Microglial ablation was performed following the paradigm described in Fig 6B. Staining for the major retinal cell types shows that overall retinal histology appears largely normal in ablated retinas. The following antibodies were used: CHX10 for bipolar cells; AP2α for amacrine cells; ChAT for starburst amacrine cells (also shows sublaminar integrity of inner plexiform layer); RBPMS for RGCs; and Sox9 for Müller glia and astrocytes. Hoechst served as nuclear counterstain. Error bars, mean ± SEM. Sample sizes are denoted by data points on graphs. For data plotted in graphs, see S1 Data. Scale bars, 50 μm. ChAT, choline acetyltransferase; DT, diphtheria toxin; DTR, diphtheria toxin receptor; GFP, green fluorescent protein; RGC, retinal ganglion cell; RNFL, retinal nerve fiber layer; TMX, tamoxifen.
(TIF)

S6 Fig Ablation of microglia using an inducible DTR system increases astrocyte number. (**A**) Two microglia-selective markers, *Cx3cr1*^*CreER-ires-YFP*^ and Iba-1, confirm absence of retinal microglia in diphtheria toxin ablated animals. The field of view was chosen to show a single microglial cell that escaped DT-mediated ablation. This cell is co-stained by antibodies to GFP and Iba-1; however, no other cells in the field of view are positive for either marker. Ablation was via the TMX/diphtheria toxin paradigm described in Fig 6B. Note that a third marker, CD45, also confirmed absence of microglia (see Fig 7E; S8E Fig). (**B**) No difference in astrocyte number between control mice that received diphtheria toxin injections (red) and those that did not receive toxin (gray). In early experiments, our breeding strategy was such that all mice in the litter inherited both CreER and DTR transgenes. In these cases, the control mice received TMX but not diphtheria toxin (except in two cases, in which control animals received neither TMX or diphtheria toxin—data for these animals are denoted by gray dots with black outlines). For subsequent experiments we changed our breeding strategy so that some mice would inherit only one of the two transgenes, allowing us to administer TMX and diphtheria toxin to all animals and still obtain unablated controls. To ask whether these two types of controls were equivalent (toxin^−^ versus toxin^+^), astrocyte numbers were compared at P8 and P10. Statistics: two-way ANOVA. There was no main effect of treatment type. Because no significant difference was found, we pooled both types of controls for subsequent analysis. (**C**) Quantification of total astrocyte numbers in control and microglia-ablated retinas. Same data as in Fig 6E; here, the data are plotted normalized to control values to highlight the magnitude of astrocyte number excess at each age. Statistics: two-way ANOVA followed by Holm-Sidak multiple comparisons test. (**D**) Ablation of microglia using transgenic *Cx3cr1-Cre* mice (“Cre line”) to drive DTR expression. Microglia were ablated following administration of diphtheria toxin (red arrows) at indicated times (top). Ablated mice carried both Cx3cr1-cre and DTR transgenes; littermate controls lacked one of the transgenes. Statistics: two-tailed *t* tests. *p*-values: microglia, *p* = 0.0185; astrocytes, *p* = 0.0158. (**E,F**) Ki67 immunostaining was used to assess astrocyte proliferation following microglia ablation (TMX/diphtheria toxin paradigm described in Fig 6B). (E) Representative images of Ki67^+^ cells in RNFL at P6. Examples of Ki67^+^/Sox9^+^ astrocytes are shown (arrows). (F) Quantification of astrocyte proliferation at P6 and P8. Microglia ablation does not affect the percentage of Ki67^+^ proliferating astrocytes. Statistics: two-way ANOVA. There was no main effect of treatment on astrocyte proliferation (see S1 Table for ANOVA details). Error bars, mean ± SEM. Sample sizes are denoted by data points on graphs. For data plotted in graphs, see S1 Data. Scale bars, 50 μm (A); 10 μm (E). DT, diphtheria toxin; DTR, diphtheria toxin receptor; GFP, green fluorescent protein; RNFL, retinal nerve fiber layer; TMX, tamoxifen.
(TIF)

S7 Fig Functional impacts of developmental absence of microglia. (**A,B**) Excess astrocytes incorporate into the astrocyte mosaic, as shown by Voronoi domain regularity analysis. (A) Representative Voronoi domains for control and diphtheria toxin ablated retinas at P10. Voronoi domains show the set of points nearest to each astrocyte within the cellular array. Cell types that show local cell–cell repulsion, such as astrocytes, will have Voronoi domains of fairly uniform size, as is the case for the control astrocyte array (left). In ablated animals, Voronoi domains are smaller due to the increased number of astrocytes. However, the size of the domains is still fairly uniform, suggesting that the increased cell number has not affected array regularity. (B) Voronoi domain regularity indices (i.e., mean/SD of the domain areas) were calculated for the astrocyte arrays in control and ablated animals. Points, individual measurements; shading denotes different animals. There is no change in regularity between the two groups. If excess astrocytes in ablated animals were dead, we would expect they would be randomly distributed and thus would lower the regularity index. As this was not observed, we conclude the excess astrocytes are incorporated normally into the nonrandom mosaic pattern. Statistics: two-tailed *t* test. **(C**) An additional example of a representative electron micrograph from P6 *Csf1r* mutant retina. Red arrows indicate examples of extravascular RBCs that have accumulated in the NFL-GCL region, indicative of bleeding. White asterisks = RGCs. (**D**) Electron micrograph from P6 *Csf1r* mutant retina. Red arrows indicate representative examples of astrocyte nuclear morphology. Astrocyte nuclei appear normal in the absence of microglia (compare with S3B Fig for wild-type examples). Analysis was performed on two sections from each of four animals; overall, 98 images were analyzed, each of which typically contained 1–2 astrocytes and occasionally contained 3 or more astrocytes. Yellow arrows indicate examples of electron-dense structures that are likely phagosomes [63]. One phagosome is within an astrocyte; the other is likely within a Müller or progenitor cell (as inferred by its radial processes). Phagosomes were not observed in astrocytes of wild-type retina (see S3B Fig). White asterisks = RBCs. (**E**) Thin plastic sections of P14 *Csf1r* null mutant retina and littermate control, viewed by light microscopy. Similar to P6 (Fig 6H), bleeding is evident in mutants. RBCs (arrows) accumulate extravascularly in the mutant NFL-GCL region. In the wild type, RBCs are confined to vasculature. (**F**) Overall anatomy of CD31^+^ retinal vasculature at P2 in control and *Csf1r* null retinas. Consistent with previous reports [60], animals lacking microglia have somewhat reduced branching frequency at P2. These are not maintained at later ages [60]. Error bars, mean ± SD. For data plotted in graphs, see S1 Data. Scale bars, 5 μm (D); 10 μm (C); 25 μm (E); 100 μm (F). GCL, ganglion cell layer; NFL, nerve fiber layer; RBC, red blood cell; RGC, retinal ganglion cell.
(TIF)

S8 Fig Assessment of compensatory mechanisms driving astrocyte death. (**A**) Temporal dynamics of astrocyte density in *Csf1r* mutants (red) and littermate controls (black). In controls, a rising phase (due to cell addition; see Fig 1A) is followed immediately by a decline phase due to microglia-mediated cell death. Mutant curves are right-shifted due to delayed onset of decline phase—i.e., delayed onset of compensatory non-microglial death. Decline phase dynamics are similar between genotypes (also see [B]). Curves were compared by nonlinear regression analysis (see [B] for details). (**B**) Nonlinear regression analysis of the *Csf1r* mutant and littermate control astrocyte density curves shown in (A). The decline phase of each curve from each regional data set was modeled (i.e., Central: P2–P14 [control], P4–P14 [null]; Middle: P4–P14 [control], P6–P14 [null]; Peripheral: P6–P14 [control and null]). A one-phase exponential decay model best fit the Central and Middle curves, while a second order polynomial (quadratic) model best fit Peripheral curves. Model parameters were similar for each mutant-control pair (Central: τ = 2.789 control, τ = 2.331 mutant; Middle: τ = 3.947 control, τ = 3.237 mutant; Peripheral: B0 = 2,066, B1 = 95.04, B2 = −14.13 control; B0 = 2089, B1 = 141.4, B2 = −13.59 mutant). (**C**) Quantification of total RGC numbers at P6 in control and *Csf1r* null retinas. RGC numbers are unchanged in the absence of microglia. This suggests that RGC death proceeds normally in *Csf1r* mutants and that loss of microglia cannot rescue them from death. Furthermore, it suggests that increased CC3^+^ nuclei (Fig 7D) are a result of corpse accumulation rather than excessive RGC death. Statistics: two-tailed *t* test (*p* = 0.2835). (**D**) CC3^+^ astrocyte density at P10 or P14 in diphtheria toxin ablated or *Csf1r* mutant retinas. Each ablation paradigm was compared to its respective littermate controls. Density of CC3^+^ astrocytes was not increased in either paradigm at these time points, further supporting the conclusion from Fig 7B that absence of microglia does not increase astrocyte apoptosis rates. Statistics: two-tailed *t* test (*p*-values: diphtheria toxin = 0.6396; *Csf1r* = 0.6728). (**E**) Representative images of CD45^+^ cells at P8 in control and diphtheria toxin ablated retinas. Images similar to these were used for quantification shown in Fig 7E. White arrow, GFP^+^/CD45^+^ microglia; yellow arrow, GFP^–^/CD45^+^ leukocyte. Error bars, mean ± SEM. Sample sizes denoted by data points on graphs (note that in [A], “Central” graph is missing one control data point located outside of y-range). For data underlying graphs, see S1 Data. Scale bar, 10 μm. CC3, cleaved-caspase 3; DT, diphtheria toxin; GFP, green fluorescent protein; RGC, retinal ganglion cell.
(TIF)

S9 Fig Astrocyte engulfment in *Csf1r* mutants. (**A**) Three-dimensional reconstruction of the Z-stack used for the image shown in Fig 7C. The large panel is identical to Fig 7C. Small panels are orthogonal views of slices through the pyknotic nucleus indicated by the arrow. Note that PDGFRα^+^ astrocyte processes (green) surround the pyknotic nucleus on all sides. (**B**) Additional example of Pax2^+^ astrocyte debris within astrocytes in *Csf1r* mutants. Arrow indicates a debris particle at the site of orthogonal slices. (**C**) Modeling of astrocyte clearance rate in *Csf1r* mutant mice, based on the frequency of astrocytes containing Pax2 debris (see Methods). For data plotted in graphs, see S1 Data. Scale bars, 5 μm (orthogonal views); 10 μm (en face views). PDGFR, platelet-derived growth factor receptor.
(TIF)

S1 Table ANOVA details. A Microsoft Excel spreadsheet providing further details on the ANOVA tests used in this study.
(XLSX)

S1 Data Numerical values for graphs. A Microsoft Excel spreadsheet containing the data plotted in each graph from all main and Supporting information figures. Each tab lists the data for several graphs, as indicated by the tab title.
(XLSX)

S1 Movie Astrocyte debris within microglia. Three-dimensional reconstruction of the confocal Z-stack depicted in Fig 4A. Microglia (green) contain Pax2^**+**^ astrocyte debris (red; yellow in overlay). Reconstruction shows that debris is genuinely located inside the microglial cells. Microglia labeled by anti-GFP in *Cx3cr1*^*CreER*^ animals.
(AVI)

S2 Movie Partial enclosure of astrocytes by microglial cells. Three-dimensional reconstruction of a confocal Z-stack showing microglia (anti-GFP, green) interacting with astrocytes (Pax2, red). At the center of the image, an astrocyte nucleus is partially enclosed by numerous arbors from a microglial cell. At the bottom of the image (at right in the first frame), a microglial cell partially encloses two astrocytes; this cell also contains Pax2^**+**^ debris (yellow). GFP, green fluorescent protein.
(AVI)

S3 Movie Extensive contact between microglial cells and astrocytes. Three-dimensional reconstruction of a confocal Z-stack showing three microglial cells (anti-GFP, green) interacting extensively with multiple astrocyes. For the purposes of Fig 4D, these interactions were largely scored as “touching.” GFP, green fluorescent protein.
(AVI)

## Abbreviations

CC3: cleaved-caspase 3
ChAT: choline acetyltransferase
CNS: central nervous system
DTR: diphtheria toxin receptor
GCL: ganglion cell layer
GFAP: glial fibrillary acidic protein
GFP: green fluorescent protein
HB-EGF: heparin binding epidermal growth factor-like factor
MMRRC: mutant mouse regional resource center
PDGFR: platelet-derived growth factor receptor
PFA: paraformaldehyde
RBC: red blood cell
RFP: red fluorescent protein
RGC: retinal ganglion cell
RNFL: retinal nerve fiber layer
VEGF: vascular endothelial growth factor
YFP: yellow fluorescent protein
