# Supplementary figures and images for: Large-scale death of retinal astrocytes during normal development is non-apoptotic and implemented by microglia
Source: PLoS Biol. 2019 Oct 18;17(10):e3000492. doi: 10.1371/journal.pbio.3000492 (PMC6821132; doi:10.1371/journal.pbio.3000492)

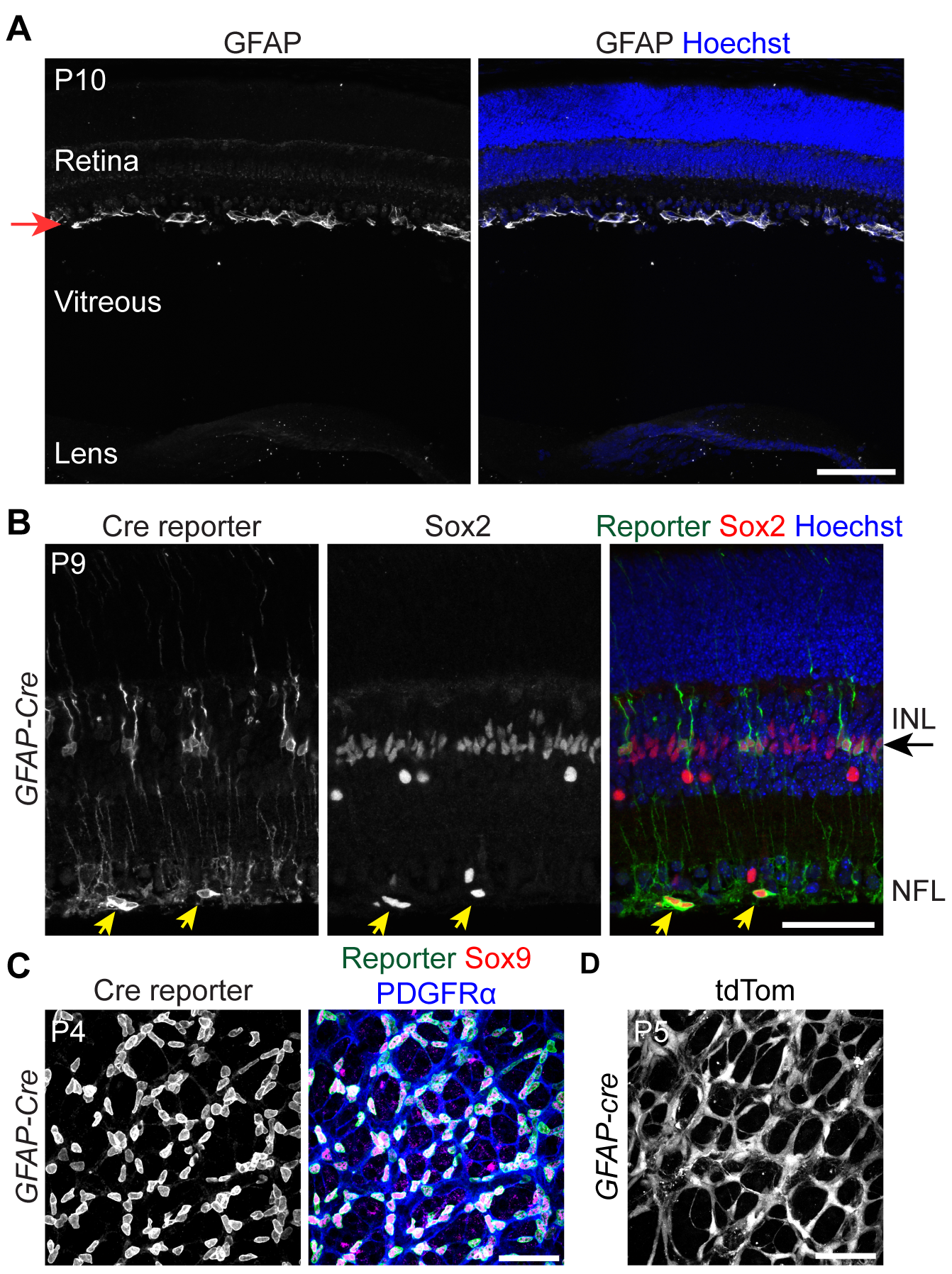

Supplement: S1 Fig — (A) Astrocytes do not migrate out of the RNFL during the death period. Whole-eye cross section from P10 mouse, stained for GFAP to label astrocytes and counterstained with Hoechst. Astrocytes are only found within the retina (in the RNFL; red arrow) and have not migrated into extraretinal spaces such as the vitreous or lens. P10 was chosen for this analysis because astrocyte numbers have declined substantially by this age, so if migration was a major cause of astrocyte loss, we should have seen many astrocytes in non-retinal regions by this time. (B) Astrocyte lineage tracing during the period of astrocyte loss, using GFAP-cre mice crossed to a Cre reporter (Rosa26iDTR). P9 retinal cross sections were immunostained with anti-DTR to reveal cells that experienced Cre activity, and for Sox2 as a marker of astrocytes and Müller glia. Cre reporter expression is only found within two astrocytic cell types: Sox2+ astrocytes of the RNFL (yellow arrows) and Sox2+ Müller glia within the INL (black arrow). This finding demonstrates that GFAP+ astrocytes do not transdifferentiate into a non-astrocytic cell type. Because astrocyte precursors activate Cre expression as early as P0–P1 [30], we would expect to see reporter-positive neurons by P9 if transdifferentiation were responsible for the decline in astrocyte numbers. If RNFL astrocytes were transdifferentiating into Müller glia, we would expect to see some reporter-positive cells migrating between the RNFL and the INL, where Müller cells reside (black arrow). However, no migrating reporter-positive cells were observed. (C) Astrocyte lineage tracing using GFAP-cre and a nuclear Cre reporter (Sun1-GFP). Representative en face images are shown. At P4, the vast majority (91.37% ± 3.12%) of Sox9+ astrocytes are also GFP+ (n = 2,819 astrocytes, N = 2 animals). This result demonstrates that Cre is active in the majority of astrocytes at early stages of their differentiation, providing an important control for the P9 lineage tracing [file pbio.3000492.s001.tif]

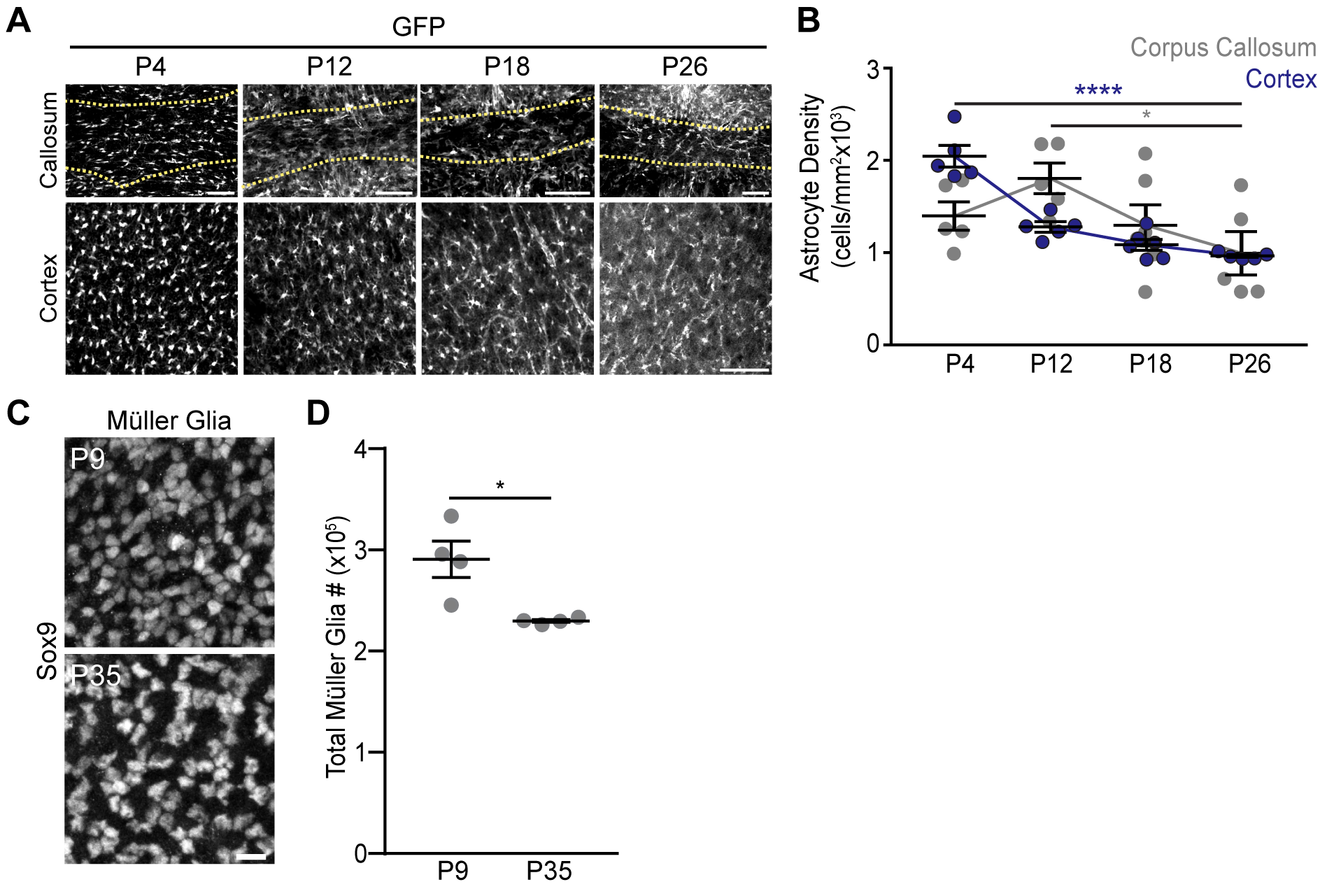

Supplement: S2 Fig — (A) Representative images of Aldh1l1-GFP+ astrocytes in corpus callosum (top) and somatosensory cortex (bottom) viewed in horizontal sections. Yellow dashed lines denote boundaries of corpus callosum. (B) Quantification of astrocyte density across development for cortex and corpus callosum. Statistics: one-way ANOVA followed by Sidak multiple comparisons test. Also see S1 Table. *p = 0.0127 (P12 versus P26); ****p <0.0001 (P4 versus P26). (C) Representative images of Müller glia nuclei, shown in en face images from retinal whole-mounts stained for Sox9. (D) Quantification of total Müller glia numbers across development (see Methods). Statistics: two-tailed t test (p = 0.0149). Error bars, mean ± SEM. Sample sizes are denoted by data points on graphs. For data plotted in graphs, see S1 Data. Scale bars, 10 μm (C); 100 μm (A). (TIF) [file pbio.3000492.s002.tif]

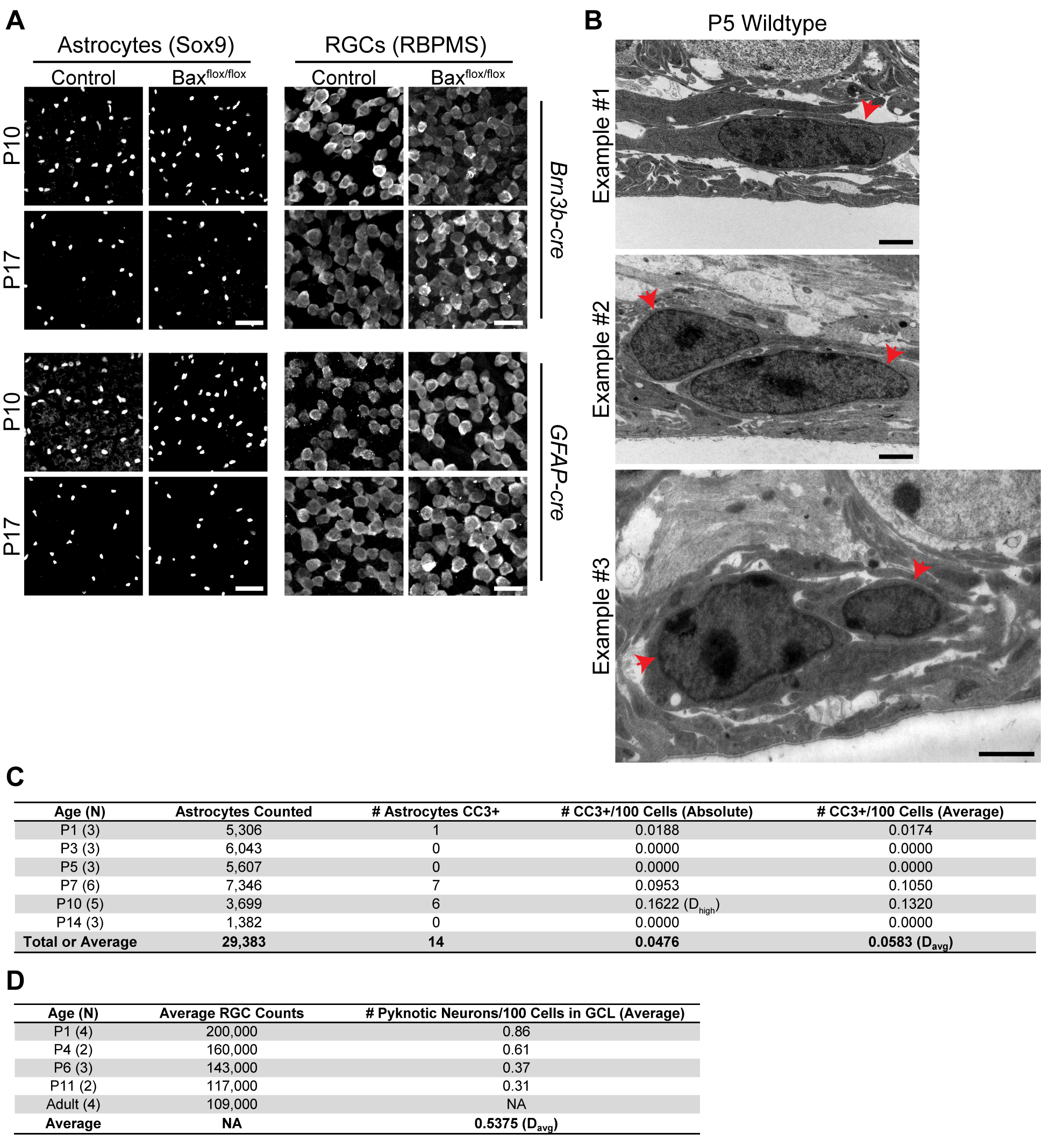

Supplement: S3 Fig — (A) Confocal images illustrating astrocyte and RGC densities in control and Bax mutant mice. Images similar to these were used for quantification shown in Fig 2D and 2E. Sox9+ astrocytes did not differ in density between wild-type controls and cell type–specific Bax mutants (left panels). More RBPMS+ RGCs are evident following Bax deletion in RGCs (Brn3b-cre) but not following Bax deletion in astrocytes (GFAP-Cre). (B) Representative electron micrographs showing ultrastructural morphology of retinal astrocytes at P5. Red arrows = astrocyte nuclei. Note the absence of anatomical features typical of various cell death pathways, such as (1) apoptosis (condensed nuclei), (2) necrosis (swollen cells/nuclei), and (3) autophagy (vacuolization) [34]. Analysis was performed on two sections from each of five animals; overall, 69 images were analyzed, each of which typically contained 1–2 astrocytes and occasionally contained >3 astrocytes. (C) Quantification of retinal astrocytes expressing CC3 across development. Davg = average death rate; Dhigh = highest death rate (both values are utilized in the model found in Fig 2C; see Methods). Overall values for the columns “Astrocytes Counted” and “# Astrocytes CC3+” are totals; overall values for the columns “#CC3/100 Cells (Absolute)” and “#CC3/100 Cells (Average)” are averages. (D) Data from Perry and colleagues (1983) quantifying rat RGCs and the number of pyknotic GCL neurons across development. These data were utilized in the model found in Fig 2B. Davg = average death rate (see Methods). Scale bars, 50 μm (A, Astrocytes); 25 μm (A, RGCs); 2 μm (B). CC3, cleaved-caspase 3; GCL, ganglion cell layer; GFAP, glial fibrillary acidic protein; RGC, retinal ganglion cell. (TIF) [file pbio.3000492.s003.tif]

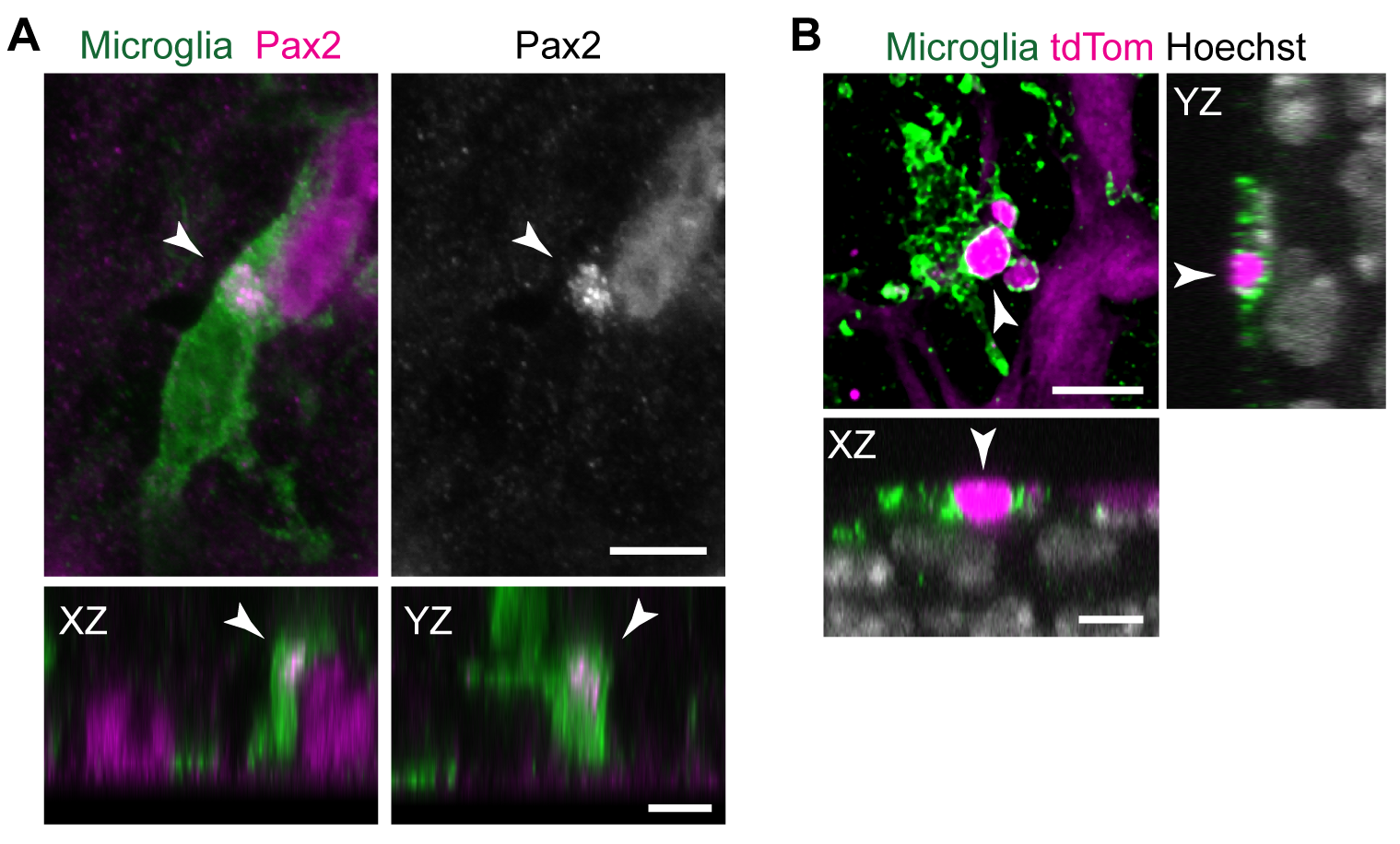

Supplement: S4 Fig — (A) An example of a Pax2+ debris punctum (arrow), similar to Fig 4A, within microglial cell labeled by Cx3Cr1CreER YFP transgene. Three-dimensional reconstruction of the confocal stack was used to generate orthogonal views (XZ and ZY) through the debris particle. From all angles, it is evident that the debris is contained within the GFP+ microglial cell. (B) The same tdTomato+ astrocyte debris shown in Fig 4B, accompanied by 3D reconstruction of confocal Z-stack. Orthogonal views through indicated debris particle (arrow) reveal that microglial phagocytic cup surrounds the debris. Scale bars, 5 μm (A, B, orthogonal views); 10 μm (B, en face view). YFP, yellow fluorescent protein. (TIF) [file pbio.3000492.s004.tif]

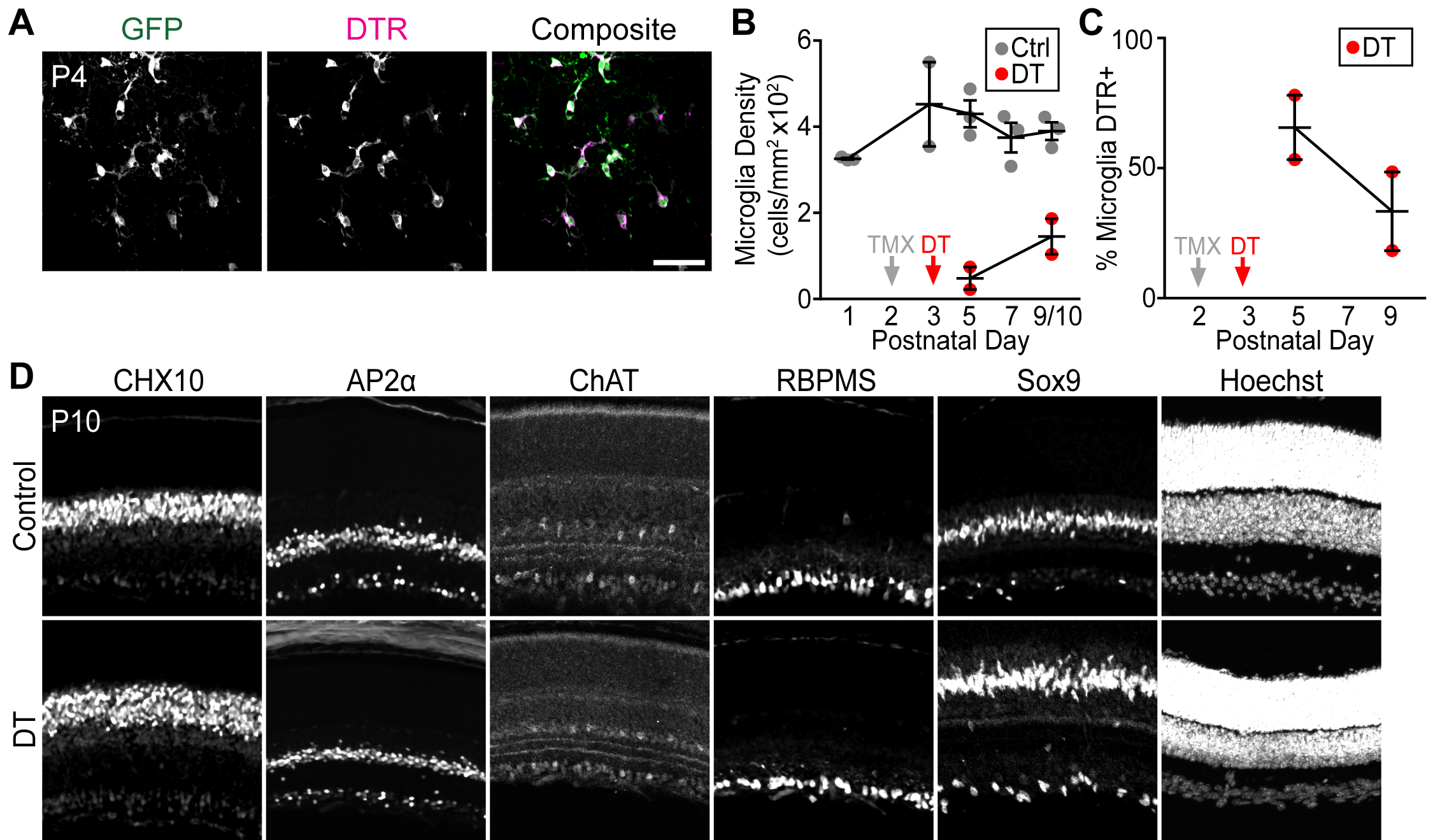

Supplement: S5 Fig — (A) Representative image of microglia from P4 Cx3cr1CreER-ires-YFP;Rosa26iDTR retina, stained for anti-GFP and anti-DTR. Mice received one dose of TMX at P2 to induce expression of DTR. Virtually all GFP+ microglia are also DTR+. See Results for cell count data. (B) Quantification of RNFL microglia density following a single round of TMX and DT, administered at the indicated time points (gray, red arrows). In Cx3cr1CreER; Rosa26iDTR animals (red data points), microglia were largely eliminated by 2 days post-toxin, but significant repopulation was seen by 4–5 days post-toxin. Based on this finding, we administered diphtheria toxin at 2-day intervals in our long-term ablation paradigm (Fig 6B). Gray data points: control data from non-littermate animals from the Cx3cr1CreER-ires-YFP background for comparison; these animals did not receive TMX or diphtheria toxin. (C) Quantification of DTR expression by spared microglia in the same ablated animals shown in (B). At 2 days post-toxin, few microglia remain (B), but a substantial fraction of these are DTR negative. The DTR-negative fraction is even higher by 6 days postinjection, suggesting that much of the repopulation is performed by microglia that escaped CreER-mediated DTR expression. This finding led us to conclude that long-term microglia ablation would require multiple TMX injections (as in the paradigm described in Fig 6B). (D) Representative retinal cross sections from P10 diphtheria toxin ablated mice or their littermate controls. Microglial ablation was performed following the paradigm described in Fig 6B. Staining for the major retinal cell types shows that overall retinal histology appears largely normal in ablated retinas. The following antibodies were used: CHX10 for bipolar cells; AP2α for amacrine cells; ChAT for starburst amacrine cells (also shows sublaminar integrity of inner plexiform layer); RBPMS for RGCs; and Sox9 for Müller glia and astrocytes. Hoechst served as nuclear counterstain. Error bars, mea [file pbio.3000492.s005.tif]

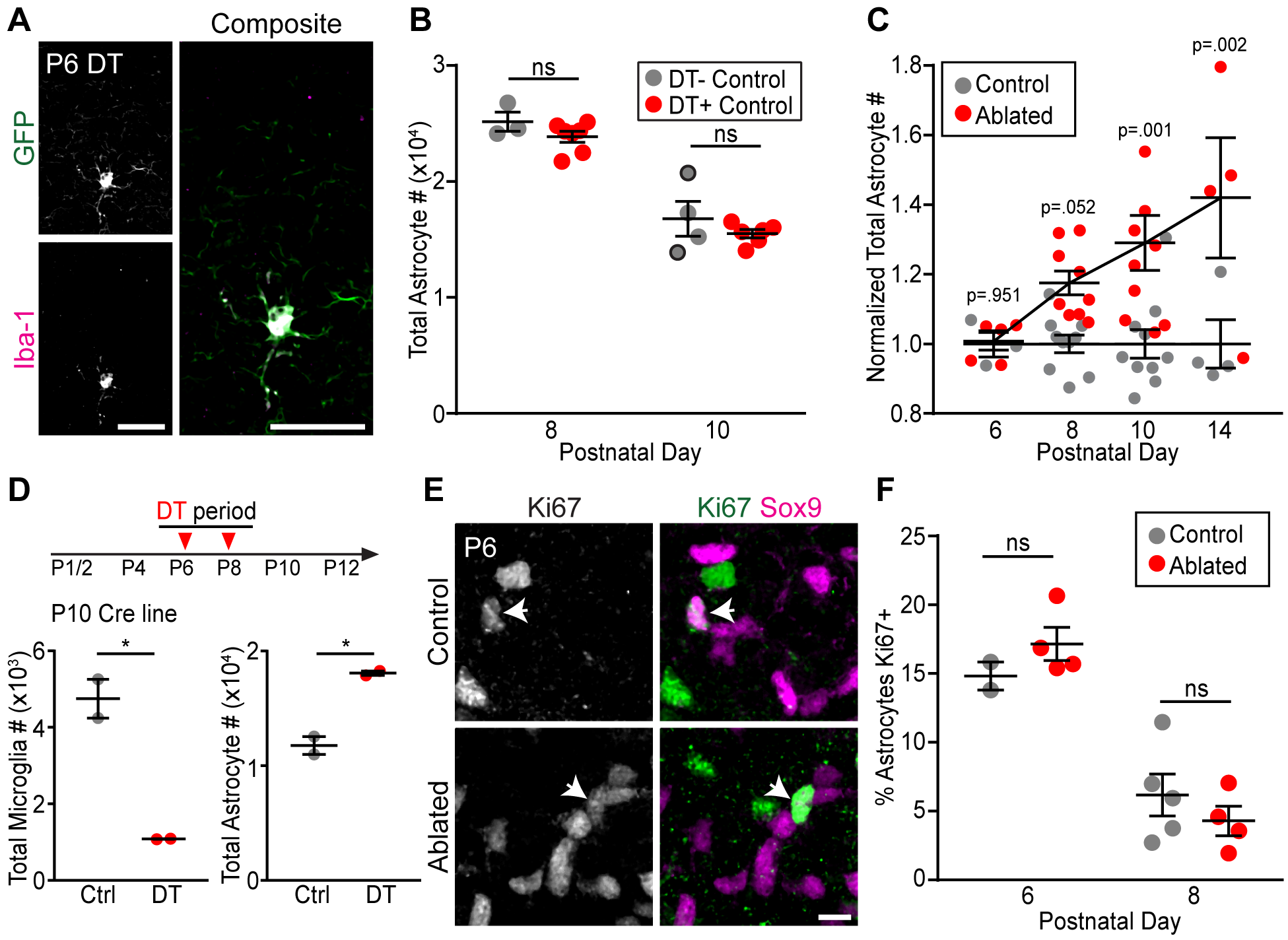

Supplement: S6 Fig — (A) Two microglia-selective markers, Cx3cr1CreER-ires-YFP and Iba-1, confirm absence of retinal microglia in diphtheria toxin ablated animals. The field of view was chosen to show a single microglial cell that escaped DT-mediated ablation. This cell is co-stained by antibodies to GFP and Iba-1; however, no other cells in the field of view are positive for either marker. Ablation was via the TMX/diphtheria toxin paradigm described in Fig 6B. Note that a third marker, CD45, also confirmed absence of microglia (see Fig 7E; S8E Fig). (B) No difference in astrocyte number between control mice that received diphtheria toxin injections (red) and those that did not receive toxin (gray). In early experiments, our breeding strategy was such that all mice in the litter inherited both CreER and DTR transgenes. In these cases, the control mice received TMX but not diphtheria toxin (except in two cases, in which control animals received neither TMX or diphtheria toxin—data for these animals are denoted by gray dots with black outlines). For subsequent experiments we changed our breeding strategy so that some mice would inherit only one of the two transgenes, allowing us to administer TMX and diphtheria toxin to all animals and still obtain unablated controls. To ask whether these two types of controls were equivalent (toxin− versus toxin+), astrocyte numbers were compared at P8 and P10. Statistics: two-way ANOVA. There was no main effect of treatment type. Because no significant difference was found, we pooled both types of controls for subsequent analysis. (C) Quantification of total astrocyte numbers in control and microglia-ablated retinas. Same data as in Fig 6E; here, the data are plotted normalized to control values to highlight the magnitude of astrocyte number excess at each age. Statistics: two-way ANOVA followed by Holm-Sidak multiple comparisons test. (D) Ablation of microglia using transgenic Cx3cr1-Cre mice (“Cre line”) to drive DTR expression. Microglia were ablated [file pbio.3000492.s006.tif]

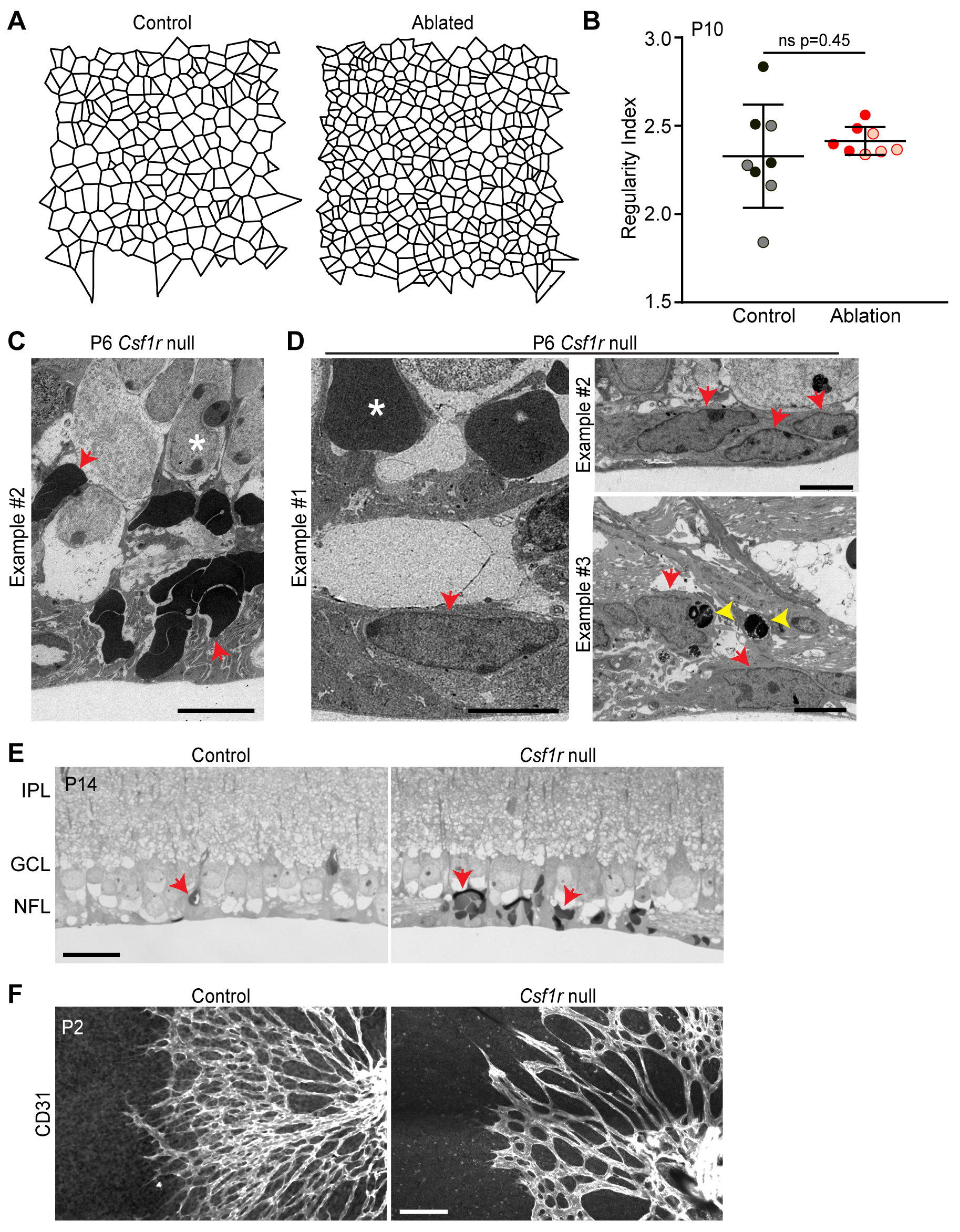

Supplement: S7 Fig — (A,B) Excess astrocytes incorporate into the astrocyte mosaic, as shown by Voronoi domain regularity analysis. (A) Representative Voronoi domains for control and diphtheria toxin ablated retinas at P10. Voronoi domains show the set of points nearest to each astrocyte within the cellular array. Cell types that show local cell–cell repulsion, such as astrocytes, will have Voronoi domains of fairly uniform size, as is the case for the control astrocyte array (left). In ablated animals, Voronoi domains are smaller due to the increased number of astrocytes. However, the size of the domains is still fairly uniform, suggesting that the increased cell number has not affected array regularity. (B) Voronoi domain regularity indices (i.e., mean/SD of the domain areas) were calculated for the astrocyte arrays in control and ablated animals. Points, individual measurements; shading denotes different animals. There is no change in regularity between the two groups. If excess astrocytes in ablated animals were dead, we would expect they would be randomly distributed and thus would lower the regularity index. As this was not observed, we conclude the excess astrocytes are incorporated normally into the nonrandom mosaic pattern. Statistics: two-tailed t test. (C) An additional example of a representative electron micrograph from P6 Csf1r mutant retina. Red arrows indicate examples of extravascular RBCs that have accumulated in the NFL-GCL region, indicative of bleeding. White asterisks = RGCs. (D) Electron micrograph from P6 Csf1r mutant retina. Red arrows indicate representative examples of astrocyte nuclear morphology. Astrocyte nuclei appear normal in the absence of microglia (compare with S3B Fig for wild-type examples). Analysis was performed on two sections from each of four animals; overall, 98 images were analyzed, each of which typically contained 1–2 astrocytes and occasionally contained 3 or more astrocytes. Yellow arrows indicate examples of electron-dense structures tha [file pbio.3000492.s007.tif]

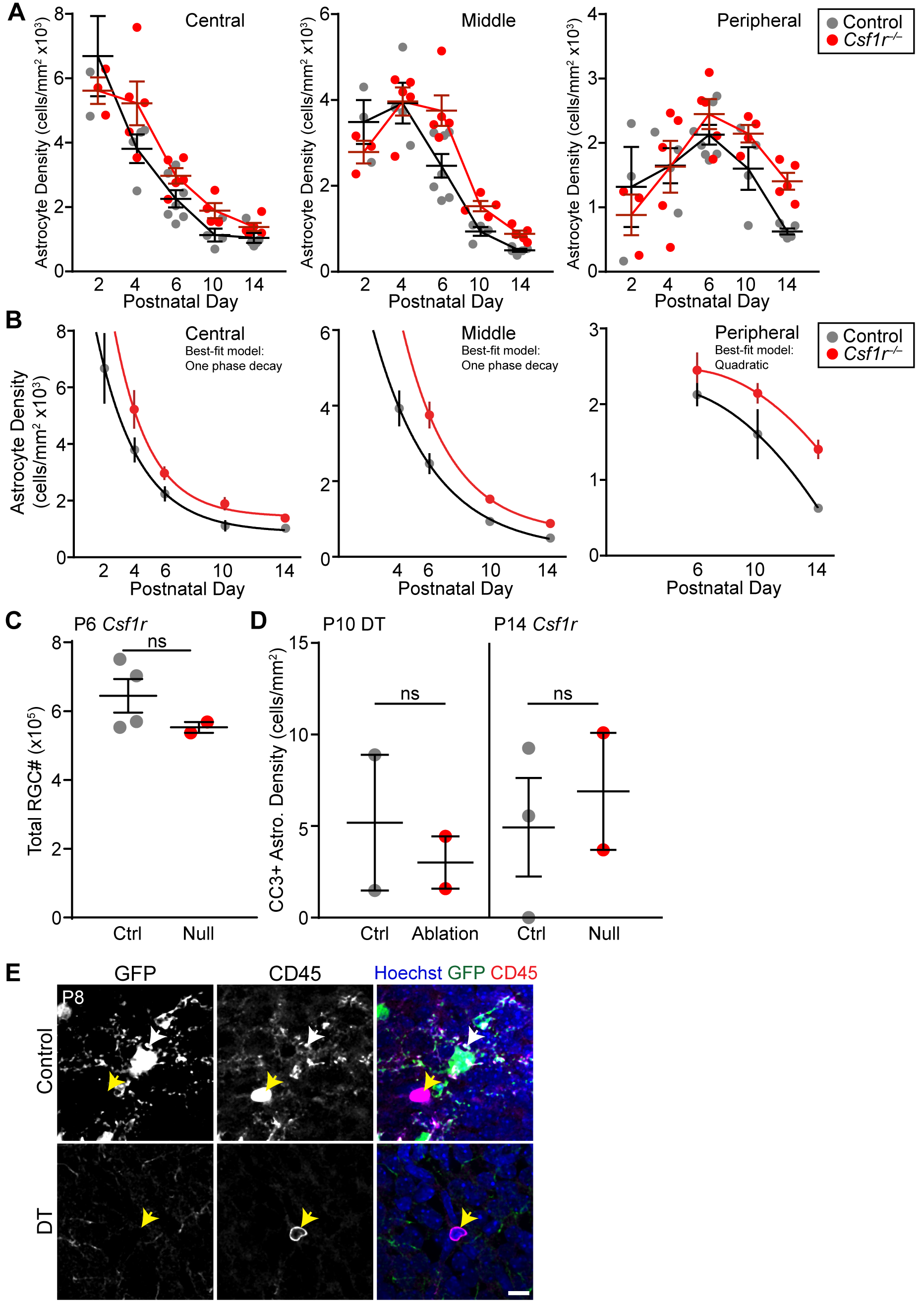

Supplement: S8 Fig — (A) Temporal dynamics of astrocyte density in Csf1r mutants (red) and littermate controls (black). In controls, a rising phase (due to cell addition; see Fig 1A) is followed immediately by a decline phase due to microglia-mediated cell death. Mutant curves are right-shifted due to delayed onset of decline phase—i.e., delayed onset of compensatory non-microglial death. Decline phase dynamics are similar between genotypes (also see [B]). Curves were compared by nonlinear regression analysis (see [B] for details). (B) Nonlinear regression analysis of the Csf1r mutant and littermate control astrocyte density curves shown in (A). The decline phase of each curve from each regional data set was modeled (i.e., Central: P2–P14 [control], P4–P14 [null]; Middle: P4–P14 [control], P6–P14 [null]; Peripheral: P6–P14 [control and null]). A one-phase exponential decay model best fit the Central and Middle curves, while a second order polynomial (quadratic) model best fit Peripheral curves. Model parameters were similar for each mutant-control pair (Central: τ = 2.789 control, τ = 2.331 mutant; Middle: τ = 3.947 control, τ = 3.237 mutant; Peripheral: B0 = 2,066, B1 = 95.04, B2 = −14.13 control; B0 = 2089, B1 = 141.4, B2 = −13.59 mutant). (C) Quantification of total RGC numbers at P6 in control and Csf1r null retinas. RGC numbers are unchanged in the absence of microglia. This suggests that RGC death proceeds normally in Csf1r mutants and that loss of microglia cannot rescue them from death. Furthermore, it suggests that increased CC3+ nuclei (Fig 7D) are a result of corpse accumulation rather than excessive RGC death. Statistics: two-tailed t test (p = 0.2835). (D) CC3+ astrocyte density at P10 or P14 in diphtheria toxin ablated or Csf1r mutant retinas. Each ablation paradigm was compared to its respective littermate controls. Density of CC3+ astrocytes was not increased in either paradigm at these time points, further supporting the conclusion from Fig 7B that absence of microglia [file pbio.3000492.s008.tif]

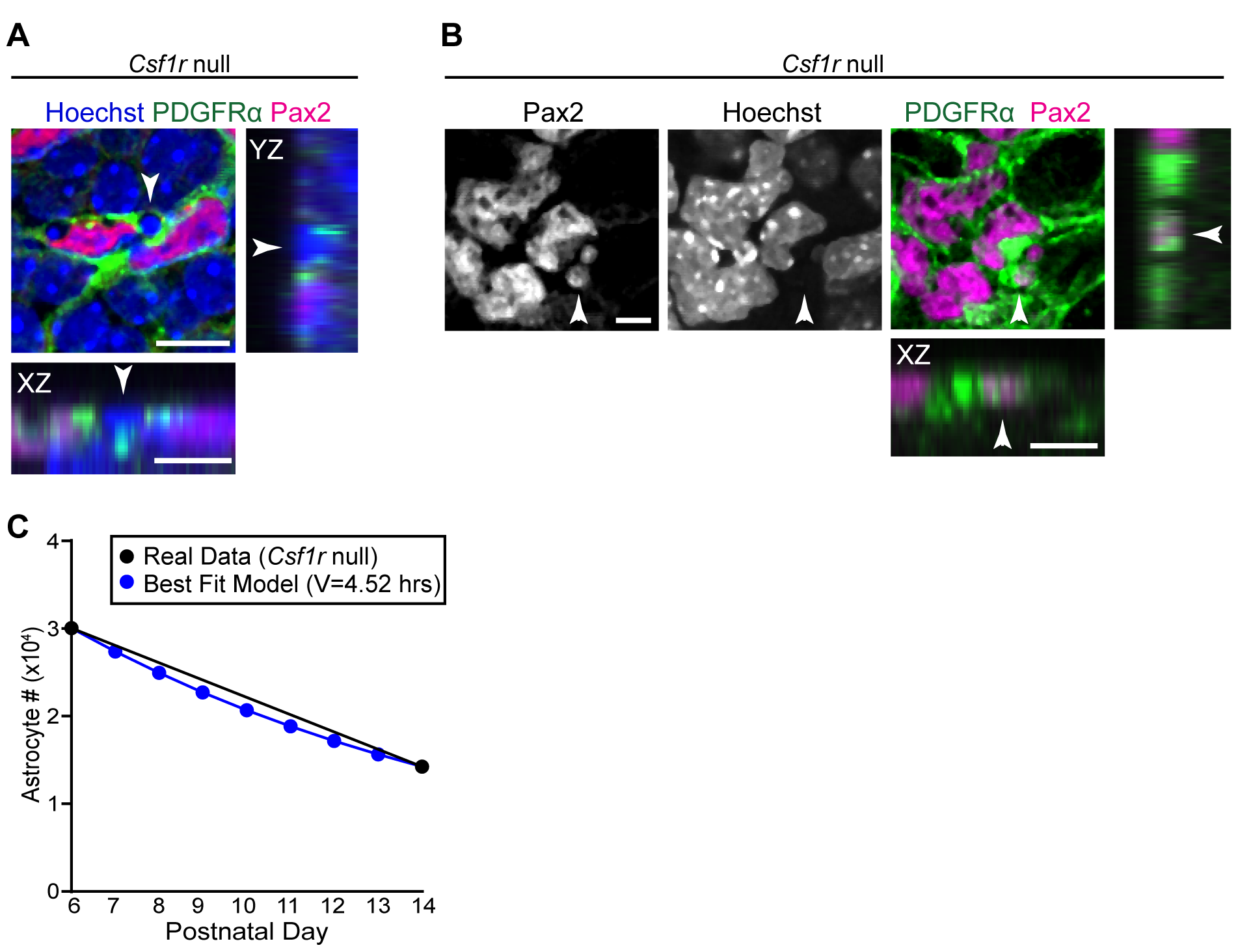

Supplement: S9 Fig — (A) Three-dimensional reconstruction of the Z-stack used for the image shown in Fig 7C. The large panel is identical to Fig 7C. Small panels are orthogonal views of slices through the pyknotic nucleus indicated by the arrow. Note that PDGFRα+ astrocyte processes (green) surround the pyknotic nucleus on all sides. (B) Additional example of Pax2+ astrocyte debris within astrocytes in Csf1r mutants. Arrow indicates a debris particle at the site of orthogonal slices. (C) Modeling of astrocyte clearance rate in Csf1r mutant mice, based on the frequency of astrocytes containing Pax2 debris (see Methods). For data plotted in graphs, see S1 Data. Scale bars, 5 μm (orthogonal views); 10 μm (en face views). PDGFR, platelet-derived growth factor receptor. (TIF) [file pbio.3000492.s009.tif]
